# Biological Overlap of Attention-Deficit/Hyperactivity Disorder and Autism Spectrum Disorder: Evidence From Copy Number Variants

**DOI:** 10.1016/j.jaac.2014.03.004

**Published:** 2014-07

**Authors:** Joanna Martin, Miriam Cooper, Marian L. Hamshere, Andrew Pocklington, Stephen W. Scherer, Lindsey Kent, Michael Gill, Michael J. Owen, Nigel Williams, Michael C. O'Donovan, Anita Thapar, Peter Holmans

**Affiliations:** a MRC Centre for Neuropsychiatric Genetics and Genomics, Institute of Psychological Medicine and Clinical Neurosciences, Cardiff University School of Medicine, UK; b Hospital for Sick Children and University of Toronto, Ontario, Canada; c Bute Medical School, University of St. Andrews, Fife, Scotland; d Trinity Centre for Health Sciences, Dublin, Ireland

**Keywords:** ADHD, ASD, pathway analysis, CNVs, comorbidity

## Abstract

**Objective:**

Attention-deficit/hyperactivity disorder (ADHD) and autism spectrum disorder (ASD) often co-occur and share genetic risks. The aim of this analysis was to determine more broadly whether ADHD and ASD share biological underpinnings.

**Method:**

We compared copy number variant (CNV) data from 727 children with ADHD and 5,081 population controls to data from 996 individuals with ASD and an independent set of 1,287 controls. Using pathway analyses, we investigated whether CNVs observed in individuals with ADHD have an impact on genes in the same biological pathways as on those observed in individuals with ASD.

**Results:**

The results suggest that the biological pathways affected by CNVs in ADHD overlap with those affected by CNVs in ASD more than would be expected by chance. Moreover, this was true even when specific CNV regions common to both disorders were excluded from the analysis. After correction for multiple testing, genes involved in 3 biological processes (nicotinic acetylcholine receptor signalling pathway, cell division, and response to drug) showed significant enrichment for case CNV hits in the combined ADHD and ASD sample.

**Conclusion:**

The results of this study indicate the presence of significant overlap of shared biological processes disrupted by large rare CNVs in children with these 2 neurodevelopmental conditions.

Attention-deficit/hyperactivity disorder (ADHD) and autism spectrum disorder (ASD) show strong comorbidity at the level of both symptoms and disorder.^1^, ^2^ Although ADHD and ASD are distinctive in terms of core diagnostic symptoms, both have onset in early childhood, present more commonly in males, and are associated with similar cognitive, developmental, and neurological problems.^3^ Twin studies have consistently shown that shared inherited factors explain a large proportion of the comorbidity between ADHD and ASD, as well as comorbidity with other neurodevelopmental problems.^4^, ^5^

Although shared common risk variants for ADHD and ASD have not been identified thus far,^6^, ^7^ this could reflect the relatively small sample sizes in the genome-wide association studies (GWAS) of each of these disorders. However, recent studies have suggested that rare (<1% frequency) chromosomal deletions and duplications, known as copy number variants (CNVs), occurring in children with ADHD show significant overlap with those already implicated in ASD.^8^, ^9^, ^10^ It is not yet known whether ADHD and ASD also more broadly share biological underpinnings.

In this study, we set out to investigate whether large rare CNVs found in individuals with each of these clinical phenotypes index disruption of shared biological pathways in the disorders. The first aim was to determine whether biological pathways disrupted by CNVs in individuals with ADHD, as compared with ethnically matched controls, showed statistically significant enrichment for CNV hits in participants with ASD, as compared to a separate set of controls. The second aim was to meta-analyze the ADHD and ASD samples to increase the power of detecting specific shared biological pathways disrupted in individuals with these 2 neurodevelopmental conditions.

## Method

### Participants With ADHD

The sample consisted of 799 young persons of white ethnicity from Cardiff, Wales (n = 559), St. Andrews, Scotland (n = 44), and Dublin, Ireland (n = 196). All children were recruited from community clinics and had a diagnosis of *DSM-IV/DSM-III-R* ADHD or International Statistical Classification of Diseases and Related Health Problems–Tenth Revision (ICD-10) hyperkinetic disorder. Exclusion criteria were intellectual disability (ID; IQ <70), major medical or neurological conditions, ASD, psychosis, and bipolar disorder. Approval was obtained from North West England, Wales, National Health Service Tayside, and Eastern Regional Health Authority research ethics committees. Written informed consent was obtained from parents, and assent/consent was gained from the young persons.

### Clinical Measures

ADHD and other psychiatric diagnoses were assessed by trained psychologists using the Child and Adolescent Psychiatric Assessment (CAPA) parent version,^11^ a semi-structured interview. Confirmation of pervasiveness of symptoms in school was obtained using the Child Attention-Deficit Hyperactivity Disorder Teacher Telephone Interview (CHATTI)^12^ or the Conner's Teacher Questionnaire.^13^ The Wechsler Intelligence Scale for Children–III/IV was used to assess IQ.^14^, ^15^ The age range was 4 through 18 years, with a mean age of 10 years 3 months (SD = 3 years). The sample was 87.4% male.

### Genome-wide Data: Individuals With ADHD and Controls

DNA for all participants with ADHD was extracted from saliva or peripheral blood samples, as described previously.^16^ Control genetic data were obtained from the Wellcome Trust Case-Control Consortium–Phase 2 (WTCCC2).^17^ Quality control (QC) procedures and CNV detection protocols were identical to those described previously.^16^ Analysis was based on single nucleotide polymorphisms (SNPs) that were present on genotyping chips in both participants with ADHD and controls. After QC, genome-wide data for 502,702 SNPs from 727 participants with ADHD and 5,081 controls were used for analysis. Analyses of CNVs were limited to those that were large (>500 kb) and rare (<1% frequency in the combined group of participants with ADHD and controls) because they have better concordance across different genotyping platforms, are determined with greater accuracy, and are more robustly associated with neurodevelopmental disorders.^8^ There were 78 large, rare CNVs within the control sample (as previously published^16^) and 85 from participants with ADHD. Parental genotype data were not available for most of the sample.

### CNV Data: Individuals With ASD

CNVs for participants with ASD and independent controls were obtained from the publicly available supplementary data of a study comparing CNVs in 996 individuals of white ethnicity with ASD to 1,287 matched controls.^18^ In this dataset, ASD diagnosis was confirmed using the Autism Diagnostic Interview–Revised (ADI-R) and Autism Diagnostic Observation Schedule (ADOS).^19^, ^20^ Control samples were obtained from the Study on Addiction: Genetics and Environment (SAGE) and from HapMap CEPH Utah (HapMap CEU).^18^, ^21^ CNVs were selected if present at <1% frequency in the total sample and having length >30kb, giving a set of 5,478 CNVs, as described previously.^18^ This CNV set contains 215 CNVs >500 kb in controls and 133 in participants with ASD. Of these 133 CNVs in participants with ASD, 13 were de novo (i.e., confirmed not to be transmitted from either parent) and 120 were confirmed to be inherited.

### Method for Testing Pathway Enrichment

#### Pathways

The following 5 sets of pathways were used in the enrichment analyses (the same as used previously^16^): Gene Ontology (GO),^22^ accessed November 8, 2011; Kyoto Encyclopedia of Genes and Genomes (KEGG),^23^ accessed June 27, 2011; PANTHER (Protein ANalysis THrough Evolutionary Relationships) pathways version 3.1,^24^ accessed February 1, 2012; Mouse Genome Informatics (MGI) database,^25^ accessed March 7, 2012; and Canonical pathways (including REACTOME and BIOCARTA) from Molecular Signatures Database (MSigDB) v3.0,^26^ accessed February 1, 2011.

For reasons of power, analyses were restricted to pathways containing between 3 and 1,500 genes (16,569 in total). Furthermore, pathways required at least 10 hits in the total sample to be counted (10,240 in total). This was to reduce the chance of small pathways showing apparent enrichment based on a small number of CNV hits.

#### Testing Pathway Enrichment of Case CNV Hits

Each CNV was assigned a binary variable (“participant” or “control”) according to whether it came from a participant with ADHD or ASD or from a control. A CNV was considered to “hit” a gene if any part of the CNV lay between the start and end points of the longest transcript of the gene (as defined by the National Center for Biotechnology Information [NCBI]). Both CNV and gene positions use build 36.3.

The following logistic regression model was fitted to the sample of CNVs for each pathway separately: *participants with ADHD or ASD/control ∼ CNV length + number of genes hit outside pathway + hit gene(s) in pathway (yes/no),* and the deviance was compared to the following: *participants with ADHD or ASD/control ∼ CNV length + number of genes hit outside pathway* to give a (1-sided) test of enrichment of case CNV hits on genes in the pathway.

This method is similar to that proposed by others^27^ and has been applied to de novo schizophrenia CNVs.^28^

*CNV length* is fitted in the model because long CNVs are more likely to hit any set of genes than small ones, and CNV length may differ systematically between participants with ADHD or ASD and controls. The *number of genes hit outside pathway* is fitted to allow for case CNVs influencing disease status by hitting genes other than those in the pathway being tested. A binary variable (yes/no) is used to indicate whether a *CNV hits gene(s) in a pathway* rather than the number of genes in the pathway hit by the CNV, to allow for some pathways having several genes that are physically close together (and thus likely to be hit by the same CNV).

The same analysis approach was also used to obtain tests of gene-specific enrichment for participant CNV hits, by defining pathways containing single genes.

#### Primary Analyses

To test whether the pathways with nominally significant enrichment in the ADHD sample showed greater than expected enrichment for case CNV hits in the ASD sample, enrichment analyses were run in the ASD sample restricted to the pathways enriched at various levels (*p* < .05, *p* < .01, *p* < .001) in the ADHD sample. The number of enriched pathways in the ASD sample at the same significance level as that used to select pathways from the ADHD sample was compared to that obtained when the participant/control labels were randomly permuted in the ASD sample. This procedure was repeated 1,000 times, and the *p* value for the number of pathways significantly enriched in both samples was estimated as the proportion of replicates, where the number of significantly enriched pathways was at least as great as that observed in the actual data. This analysis allows for overlap between pathways in terms of their gene membership. Analyses were carried out using all participants with ASD CNVs (versus control CNVs), and also for de novo and inherited CNVs (versus all control CNVs), separately.

A combined pathway enrichment analysis was performed by meta-analyzing the ADHD and ASD case CNVs compared to their corresponding control CNVs. Enrichment *p* values for both genes and pathways were obtained by adding a 2-level factor coding for sample (ADHD/ASD) to the regression models (to allow for possible differences in CNV calling and/or ethnic differences between samples) and applying these to the combined ADHD and ASD data.

#### Secondary Analyses

To assess the extent to which the observed overlap in enriched pathways is driven by known loci for autism susceptibility, the enrichment analysis was repeated on the complete pathway set, omitting genes in 14 autism loci from previous studies (Chr1 174.1–175.1 Mb, Chr2 13.12–13.16 Mb, Chr2 49.99 Mb–51.12 Mb, Chr3 2.11–3.08 Mb, Chr3 4.37–4.49 Mb, Chr3 122.83–122.87 Mb, Chr3 174.59–175.49 Mb, Chr4 144.85 Mb, Chr6 161.68–163.07 Mb, Chr7 68.69–69.88 Mb, Chr10 87.33–88.12 Mb, Chr15 23.12–23.24 Mb, Chr16 29.55–30.08 Mb, Chr22 49.44–49.52 Mb). Note that these loci have previously been shown to overlap with CNVs in a portion (n = 366) of the current ADHD sample.^8^

Moreover, it is possible that any observed overlap in pathways enriched in both samples is due to the physical overlap between case CNVs from the 2 samples, rather than shared biology per se (although larger than expected physical overlap may, of course, be due to shared biology). We tested whether ASD participants' CNVs are more likely than their control peers' CNVs to overlap participants with ADHD participants' CNVs, and vice versa, by fitting the following logistic regression model to the CNVs from each sample: *Overlap (y/n) ∼ participants with ADHD or ASD/control + CNV length.* A CNV was defined as overlapping if any part of that CNV overlapped any case CNV from the other sample. A significant positive regression coefficient for the case/control term is taken as evidence that case CNVs in 1 sample are more likely than their corresponding control CNVs to overlap case CNVs in the other sample, allowing for CNV size (large CNVs being more likely to overlap than small ones). This analysis was initially carried out genome-wide, using all CNVs, and then repeated omitting CNVs in the autism regions listed above to see if these regions accounted for any significant overlap.

The pathway enrichment analyses were repeated on the complete pathway set after removing from both sets of cases any CNV occurring in a participant with ADHD that overlapped a CNV in the ASD sample (leaving 102 ASD CNVs and 55 ADHD CNVs). A significant excess of pathways enriched in both samples would provide evidence of shared biology even among CNVs that do not hit the same genes.

To determine whether any observed overlaps in significant pathways were driven primarily by deletions or duplications, the pathway enrichment analyses were repeated on the complete pathway set, using deletions or duplications alone.

## Results

### Significant Overlap in Biological Pathways Enriched in Both Samples

Table 1 shows the number of pathways achieving differing significance levels (*p* < .05, *p* < .01, *p* < .001) in the ASD sample that were also significant at the same significance level in the ADHD sample. It can be seen that a significant overlap in enriched pathways for ASD and ADHD is observed for both ASD de novo and inherited CNVs, with the most significant overlap being observed in the analysis of all ASD CNVs together.

**Table 1:** Number of Pathways Achieving Given Levels of Enrichment Significance (*p* < .05, *p* < .01, *p* < .001) in the Autism Spectrum Disorder (ASD) Dataset That Were Also Significantly Enriched at the Same Significance Level in the Attention-Deficit/Hyperactivity Disorder (ADHD) Sample

CNV Type (ASD)	*p* < .05		*p* < .01		*p* < .001	
	No. of Pathways	*p*	No. of Pathways	*p*	No. of Pathways	*p*
De novo	58	.006	9	.016	1	.021
Inherited	72	.001	16	.004	1	.019
All	100	<.001	20	.001	1	.017

Note: *p* Values are given for the test of whether the number of enriched pathways is greater than would be expected by chance. CNV = copy number variant; de novo = confirmed not to have been transmitted from either parent.

### Enriched Pathways and Genes in the Combined ADHD and ASD Dataset

Of the 100 pathways that were significantly (*p* < .05) enriched for case CNV hits in both the ADHD and ASD samples, the 20 pathways that were most significantly enriched in the combined ADHD and ASD (all CNV) samples are shown in Table 2, together with the number of gene hits by case CNVs, and the genes that are individually significantly (nominal *p* < .05) enriched for case CNV hits in the combined ADHD and ASD sample. Note that 3 of these pathways (nicotinic acetylcholine receptor signaling, cell division, and response to drug) have enrichment *p* values in the combined ADHD and ASD data that are significant even after Bonferroni correction for 10,240 pathways tested (*p* < 4.88 × 10^−6^). This correction is conservative, as the tested pathways are not independent because of shared genes. The genes hit by case CNVs in these 3 pathways are listed in Table S1 (available online).

**Table 2:** The 20 Pathways (Ranked by Combined Attention-Deficit/Hyperactivity Disorder [ADHD] and Autism Spectrum Disorder [ASD] *p* Values) That Were Most Significantly Enriched for Individual Copy Number Variant (CNV) Hits in the Combined ADHD and ASD (All CNV) Dataset

Pathway ID	No. of Genes	No. of Gene Hits (Combined)	No. Gene Hits (ADHD)	No. of Gene Hits (ASD)	*p* (Combined)	*p* (ADHD)	*p* (ASD)	Pathway Description	Significant Genes (Combined Sample)
PAN-PW44	89	32	19	13	1.75E-07	3.39E-07	5.81E-03	Nicotinic acetylcholine receptor signaling pathway	CHRNA7, MYH11
GO:51301	364	39	20	19	7.69E-07	2.19E-04	1.64E-03	Cell division	AATF, NDE1, CHMP1B
GO:42493	322	50	28	22	8.94E-07	4.11E-04	5.69E-04	Response to drug	ACACA, ABCC6,ABCC1
GO:5516	147	21	10	11	5.66E-06	2.87E-03	9.50E-04	Calmodulin binding	MYH11
GO:5794	1,022	97	46	51	9.77E-06	2.10E-03	2.35E-03	Golgi apparatus	ABCC1, TJP1, SYNRG, PARM1, AATF, XYLT1, MPPE1
MGI:5620	212	23	15	8	1.15E-05	7.41E-04	1.82E-02	Abnormal muscle contractility	MYH11
GO:6195	625	60	31	29	1.80E-05	6.26E-03	2.20E-03	Purine nucleotide catabolic process	ABCC6, GNAL, ABCC1, DDX52
GO:9154	602	60	31	29	1.80E-05	6.26E-03	2.20E-03	Purine ribonucleotide catabolic process	ABCC6, GNAL, ABCC1, DDX52
GO:9261	604	60	31	29	1.80E-05	6.26E-03	2.20E-03	Ribonucleotide catabolic process	ABCC6, GNAL, ABCC1, DDX52
GO:9143	600	58	32	26	1.99E-05	3.75E-03	4.24E-03	Nucleoside triphosphate catabolic process	ABCC6, GNAL, ABCC1, DDX52
GO:9166	642	62	32	30	2.21E-05	9.48E-03	2.18E-03	Nucleotide catabolic process	ABCC6, GNAL, ABCC1, DDX52
GO:9141	640	64	33	31	2.48E-05	2.17E-02	9.13E-04	Nucleoside triphosphate metabolic process	ABCC6, GNAL, ABCC1, DDX52
GO:9144	631	63	32	31	2.50E-05	2.18E-02	9.13E-04	Purine nucleoside triphosphate metabolic process	ABCC6, GNAL, ABCC1, DDX52
GO:9199	628	63	32	31	2.50E-05	2.18E-02	9.13E-04	Ribonucleoside triphosphate metabolic process	ABCC6, GNAL, ABCC1, DDX52
GO:9205	627	63	32	31	2.50E-05	2.18E-02	9.13E-04	Purine ribonucleoside triphosphate metabolic process	ABCC6, GNAL, ABCC1, DDX52
PAN-PW16	69	18	10	8	2.76E-05	1.59E-04	2.56E-02	Cytoskeletal regulation by rho GTPase	MYH11
GO:6633	108	23	11	12	2.77E-05	2.16E-03	4.19E-03	Fatty acid biosynthetic process	ACACA,ABCC1
GO:48285	306	33	15	18	2.88E-05	1.59E-02	9.87E-04	organelle fission	NDE1
GO:10927	94	29	18	11	2.88E-05	3.29E-03	2.48E-03	Cellular component assembly involved in morphogenesis	MYH11, FOPNL
GO:6461	499	50	25	25	3.01E-05	8.82E-03	1.41E-03	Protein complex assembly	ACACA, MYH11

Note: Pathways are sorted in order of enrichment *p* value in ADHD and ASD combined (the *p* (Combined) Column). Bonferroni correction for 10,240 pathways corresponds to a *p* (Combined) < 4.88 × 10^−6^. The number of gene hits by case CNVs in each pathway are also shown, as are the individually significant (nominal *p* < .05) genes.

### Significant Overlap in Physical Locations of Case CNVs Between ASD and ADHD

ASD case CNVs were significantly more likely than ASD control CNVs to overlap with ADHD case CNVs (*p* = 4.77 × 10^−3^), even when 14 specific ASD susceptibility loci were excluded (*p* = 8.28 × 10^−3^). Similarly, ADHD case CNVs were significantly more likely to overlap with ASD case CNVs than ADHD control CNVs (*p* = 5.49 × 10^−4^; known regions excluded: *p* = 3.52 × 10^−4^).

### Removal of CNVs in Known ASD Regions and Overlapping Case CNVs

Secondary analyses assessed whether these observed results were driven by specific loci previously implicated in ASD and shown to overlap with CNVs in a subsample of the current ADHD sample,^8^ or by overlapping CNV regions in the 2 groups, including CNVs other than those falling within these known loci. Analyses were repeated omitting these regions. Results are shown in Table 3.

**Table 3:** Number of Pathways Achieving Given Levels of Enrichment Significance (*p* < .05, *p* < .01, *p* < .001) in the Autism Spectrum Disorder (ASD) Dataset That Were Also Significantly Enriched at the Same Significance Level in the Attention-Deficit/Hyperactivity Disorder (ADHD) Sample

CNV Type (ASD)	*p* <.05		*p* < .01		*p* < .001	
	No. of Pathways	*p*	No. of Pathways	*p*	No. of Pathways	*p*
Excluding “known” ASD regions						
De novo	52	.006	4	.073	0	1
Inherited	78	.003	16	.004	1	.018
All	98	<.001	23	.001	2	.008
Excluding all overlapping CNVs						
De novo	12	.009	3	.010	0	1
Inherited	4	.256	0	1	0	1
All	7	.130	1	.116	0	1

Note: *p* Values are given for the test of whether the number of enriched pathways is greater than would be expected by chance. Analyses exclude genes in 14 “known” autism regions, and any ASD case copy number variants (CNVs) that overlap ADHD case CNVs and vice versa (see text). De novo = confirmed not to be transmitted from either parent.

For the analysis omitting the specific regions, it can be seen that the overlap in pathways is still significant, although, not surprisingly, the level of significance is reduced, particularly for analyses based on the ASD de novo CNVs. For the analysis omitting all overlapping case CNVs, results show a modestly significant overlap of enriched pathways in the ASD de novo CNVs but not in the inherited CNVs or the total CNV set.

Pathway enrichment *p* values in the absence of CNVs overlapping known ASD loci or overlapping a case CNV from the other disorder are shown in Table S2 (available online) for all 100 pathways significantly (*p* < .05) enriched in both ADHD and ASD when all CNVs were analyzed (primary analysis). Removing the known ASD regions makes little difference to the pathway enrichment, whereas removing case CNVs that physically overlap with case CNVs from the other disorder generally reduces enrichment significance considerably. Thus, most of the overlap in enriched pathways can be attributed to case CNVs in the disorders hitting the same loci (and thus genes) but not necessarily in regions previously implicated in ASD.

### Analysis of Deletions and Duplications Separately

Of the 85 case CNVs in the ADHD sample, 21 were deletions and 64 were duplications. Among the 78 control CNVs in the ADHD dataset, 13 were deletions and 65 were duplications. Of the 133 case CNVs in the ASD dataset, 34 were deletions and 99 were duplications. Among the control CNVs in the ASD dataset, 65 were deletions and 150 were duplications. The numbers of pathways significantly enriched in both ADHD and ASD are provided in Table 4. Analyzing duplications and deletions separately reduces both the number of pathways significantly enriched in both ADHD and ASD and the significance of any excess. There was no evidence that either deletions or duplications separately account for the observed pathway overlap between ADHD and ASD. Pathway-specific enrichment *p* values for the 100 pathways significantly enriched (*p* < .05) in both ADHD and ASD when deletions and duplications are analyzed separately are shown in Table S3 (available online).

**Table 4:** Number of Pathways Achieving Given Levels of Enrichment Significance (*p* < .05, *p* < .01, *p* < .001) in the Autism Spectrum Disorder (ASD) Dataset That Were Also Nominally Significantly Enriched at the Same Significance Level in the Attention-Deficit/Hyperactivity Disorder (ADHD) Sample

CNV Type (ASD)	*p* < .05		*p* < .01		*p* < .001	
	No. of Pathways	*p*	No. of Pathways	*p*	No. of Pathways	*p*
All	100	<.001	20	.001	1	.017
Deletions	1	.153	0	1	0	1
Duplications	41	.032	2	.203	0	1

Note: *p* Values are given for the test of whether the number of enriched pathways is greater than would be expected by chance. Analyses are shown for all copy number variants (CNVs) and also for deletions and duplications separately.

### List of CNVs Used in the Analyses

A complete list of the case CNVs >500 kb (ADHD and ASD) used in the analyses is given in Table S4 (available online).

## Discussion

The results show that the biological pathways enriched (*p* < .05) for CNVs in the ADHD sample (relative to controls) as a group show more enrichment for CNVs in the ASD sample (relative to an independent set of controls) than expected by chance. A similar enrichment was observed (results not shown) when the analyses were performed in the reverse direction (i.e., the pathways enriched in the ASD sample tested in the ADHD sample). This finding indicates the presence of common biological liability for ADHD and ASD. Significant overlap was observed for both de novo and inherited CNVs in the ASD sample, although these results are not independent because the same control CNVs were used.

Given that an earlier study using part of the current ADHD sample found enrichment in ADHD CNV loci that had been previously implicated in ASD,^8^ those loci were omitted from the current analysis to obtain independent replication of the earlier finding that ASD CNV loci were also found in children with ADHD. The continued significant overlap of CNVs and pathways suggests that other CNV loci are also contributing to this effect, although, given the small number of loci in this additional analysis, the significance of overlap with ADHD and de novo ASD CNVs is reduced. Moreover, omitting all ADHD case CNVs overlapping at all with ASD case CNVs and vice versa from the analyses also shows more generally that shared biological pathways are implicated in ADHD and ASD above and beyond overlap of specific CNV regions. Given that performing this strict analysis with no physically overlapping CNVs substantially reduces the pool of CNVs in the analysis, it is remarkable that there is still demonstrable overlap in biological pathways tapped into by CNVs from participants with ADHD and ASD.

To highlight which specific pathways contain enrichment evidence in both ADHD and ASD, the 2 samples were combined in a joint analysis. Three pathways showed significant enrichment after correction for multiple testing (“nicotinic acetylcholine receptor signalling pathway,” “cell division,” and “response to drug”). Owing to the definition of pathway categories, many of the analyzed pathways overlap with one another, including pathways embedded in one another. The 3 significant pathways contain different significant genes (Table 2 and Table S1; the latter is available online) despite each pathway containing at least 1 significant gene from the same region on chromosome 16 (*MYH11, NDE1, ABCC1, ABCC6*), and a significant (or nearly significant) gene from the same region on chromosome 17 (*MYO19, AATF, ACACA*). It is unclear which gene(s) in these regions is responsible for the CNV enrichment. It should be noted that even when these regions are removed from the analysis, all 3 pathways still show significant enrichment (nicotinic acetylcholine receptor signaling pathway: *p* = 1.17 × 10^−3^, cell division: *p* = 6.06 × 10^−3^, response to drug: *p* = 3.41 × 10^−3^).

It is interesting to note that the neurobiology encompassed by these pathways enriched in both ADHD and ASD has been implicated in a previous pathway analysis of the current ADHD sample, which explored the overlap of common (SNPs) and rare (CNVs) variants.^16^ The most significantly enriched pathway in the combined ADHD and ASD samples (Table 2) is the PANTHER pathway “nicotinic acetylcholine receptor signalling,” which is also significantly enriched in both ADHD and ASD separately. This pathway contains 2 genes, *MYH11* and *CHRNA7,* of potential interest. The gene *CHRNA7* encodes the alpha 7 nicotinic acetylcholine receptor, which has a role in calcium signaling in the brain. This gene was previously shown to be enriched for both CNV hits and GWAS signal in the current ADHD sample.^16^ It has been found also to have duplications spanning it in a genome-wide analysis of CNVs, a finding that was replicated in independent ADHD samples.^10^ *CHRNA7* is located at the chromosomal locus of 15q13.3, and deletions at this locus have been found to be associated with different neurodevelopmental abnormalities and neuropsychiatric disorders.^29^ There is evidence from a small case series that deletions and duplications at this locus could also be associated with features of ASD.^30^ *MYH11* is at the chromosomal locus of 16p13.1, a region that has previously been shown to be enriched for CNVs in a subsample of participants with ADHD, at a genome-wide level relative to controls.^8^ This region has also been implicated in autism,^31^ schizophrenia,^32^ and intellectual disability/multiple congenital anomalies.^33^ It should be noted that *MYH11* is involved in numerous other enriched pathways, suggesting that it may influence ADHD and ASD susceptibility through multiple biological processes.

Interestingly, the current analysis has not implicated the types of biological pathways previously reported in pathway analyses in ASD samples that are related to synaptic and neuronal plasticity and those involved in neurotransmission or synapse formation and maintenance.^34^, ^35^ This is likely because such pathways are not significantly enriched for case CNV hits in the ADHD sample.

Given the very high level of comorbidity and symptom correlation between ADHD and ASD,^1^ it is arguable as to whether it is clinically meaningful to attempt to distinguish “pure” ASD or ADHD cases. Although children with a clinician's diagnosis of ASD were excluded from the ADHD sample, previous clinical analyses have reported subthreshold ASD traits in this sample.^36^ Moreover, clinical data on levels of ADHD symptoms were not available for the ASD cases for this analysis.^18^ Thus, it is possible that the overlap in biological pathways detected in this study may be reflecting the presence of subthreshold ADHD and ASD traits in the samples. However, given the strong relationship of the 2 conditions, the value of attempting to control for subthreshold traits is unclear. Also, despite overlaps, ADHD and ASD are clinically distinctive. Furthermore, disruption of synaptic function by rare CNVs may play a more important role in some neurodevelopmental processes, such as those involved in ASD and ID, but not all. Thus far, synaptic functions have not been implicated by pathway analysis of SNP data in ADHD.^16^

Although the participants with ADHD and the WTCCC2 controls were genotyped on different chips, CNV calling used only the SNPs common to both genotyping chips, with the same QC procedures used to filter the SNPs. Thus, the CNV calls are comparable between participants with ADHD or ASD and controls, particularly for large (>500-kb) CNVs that are called with high accuracy and reliability. The participants with ASD and controls were genotyped on the same chips, using the same QC protocols, so again the CNV calls are directly comparable between participants with ASD and controls. The participants with ADHD and WTCCC controls are of similar ethnic backgrounds (UK individuals of white ethnicity), as are the participants with ASD and controls (US white individuals). Note that, in the combined analysis, differences in ethnicity and CNV calling between the ADHD and ASD samples are controlled by including “sample” as a covariate. Thus, significant pathway enrichments are unlikely to have arisen because of population stratification or differences in CNV calling.

One limitation of this study is that the biological pathway categories were defined based on the GO, KEGG, PANTHER, MGI, and MSigDB databases, which depend on the accuracy of the annotations in these databases. As the contents of these databases come to reflect the growing knowledge of these processes, pathway analyses such as those described in this article will be better able to implicate specific biological processes in disease etiology. Furthermore, future studies need to assess the functional effects of genes implicated in specific overlapping pathways, to determine the exact nature and extent of the shared underlying biology of ADHD and ASD.

It is of note that the overlap in pathways was detected for de novo as well as inherited CNVs. Both inherited and de novo CNVs are enriched in participants with ASD relative to controls, with a higher frequency of de novo CNVs in females than in males and in sporadic ASD cases (i.e., families with a single affected child) than in “multiplex” families (i.e., with more than 1 affected child).^37^, ^38^, ^39^, ^40^ De novo CNVs have also been reported in individuals with ADHD, although at a lower rate than that reported in ASD and schizophrenia.^9^ Unfortunately, because of the unavailability of complete parental genetic data, it is not known whether the CNVs in the ADHD sample were inherited or de novo.

Although the majority of the CNVs in this sample (both in participants with ADHD or ASD and in controls) were duplications, the strength of the signal was reduced by analyzing deletions and duplications separately. This suggests that both types of CNVs likely contribute to disrupting biological processes underlying ADHD and ASD.

There is evidence that large, rare CNVs are more likely to occur in children with ADHD who have comorbid ID (IQ <70).^8^ Similarly, there is a somewhat greater rate of CNVs in children with ASD with ID relative to those without ID.^41^ Although children with ID were excluded from the ADHD analyses, the ASD sample did not make such exclusions. However, this would serve to reduce biological overlap, and it means that the results cannot be explained by comorbid ID.

In conclusion, this study provides evidence that ADHD and ASD show significant overlap of shared biological processes being disrupted by CNVs. This finding gives preliminary evidence of the mechanisms that may underpin observed phenotypic overlap^1^ and shared heritability.^4^ The findings would benefit from replication and further investigation using larger collaborative samples and future updated versions of pathway annotation databases to determine the specific biological pathways that are being affected by these rare variants. These findings further strengthen the conceptual grouping of ADHD and ASD as related neurodevelopmental disorders. Further research in this area has the potential to shed light on heterogeneity of ADHD and ASD clinical phenotypes and the subtyping of child neurodevelopmental disorders.

## Figures and Tables

**Table S1:** Genes in the 3 Pathways Significantly Enriched in the Combined Attention-Deficit/Hyperactivity Disorder (ADHD) and Autism Spectrum Disorder (ASD) Dataset, After Multiple Testing

Pathway	Entrez ID	Gene Symbol	Chr	Start	End	No. of Hits (Combined Cases)	*p* (Combined)
GO:42493	4363	ABCC1	16	15950935	16144432	11	2.22E-05
GO:51301	54820	NDE1	16	15651605	15726491	11	2.22E-05
PAN-PW44	4629	MYH11	16	15704493	15858388	11	2.22E-05
GO:42493	368	ABCC6	16	16150923	16224838	11	3.66E-04
PAN-PW44	1139	CHRNA7	15	30110018	30248541	11	1.35E-02
GO:51301	57132	CHMP1B	18	11841426	11842697	2	2.27E-02
GO:51301	26574	AATF	17	32380288	32488284	3	2.82E-02
GO:42493	31	ACACA	17	32516040	32841015	3	2.82E-02
PAN-PW44	80179	MYO19	17	31925712	31964838	2	6.11E-02
GO:51301	4851	NOTCH1	9	138508717	138560059	2	6.90E-02
GO:51301	84861	KLHL22	22	19125806	19180122	4	7.53E-02
PAN-PW44	9342	SNAP29	22	19543292	19574109	4	7.53E-02
GO:42493	9420	CYP7B1	8	65671246	65873902	1	7.75E-02
GO:51301	1070	CETN3	5	89725284	89741359	1	7.75E-02
GO:42493	7436	VLDLR	9	2611793	2644485	1	7.79E-02
GO:51301	25909	AHCTF1	1	245069023	245148302	1	7.85E-02
GO:42493	2571	GAD1	2	171381446	171425907	1	8.08E-02
GO:42493	65985	AACS	12	124115878	124193824	1	8.40E-02
GO:51301	389	RHOC	1	113045272	113051548	1	8.45E-02
GO:42493	389	RHOC	1	113045272	113051548	1	8.45E-02
GO:42493	58189	WFDC1	16	82885902	82920951	1	8.54E-02
GO:42493	5174	PDZK1	1	144439083	144475430	1	9.41E-02
GO:42493	11280	SCN11A	3	38862264	38967056	1	9.49E-02
GO:51301	29945	ANAPC4	4	24987946	25029218	1	1.19E-01
GO:42493	9429	ABCG2	4	89230440	89299035	1	1.27E-01
PAN-PW44	71	ACTG1	17	77091594	77094422	1	1.28E-01
GO:51301	51529	ANAPC11	17	77442895	77451655	1	1.28E-01
GO:42493	1374	CPT1A	11	68278664	68365881	1	1.28E-01
GO:42493	51083	GAL	11	68208559	68215219	1	1.28E-01
GO:51301	151011	SEPT-10	2	109657665	109729072	1	1.29E-01
GO:51301	10015	PDCD6IP	3	33814561	33886198	1	1.30E-01
GO:51301	23122	CLASP2	3	33512741	33734852	1	1.30E-01
GO:51301	23310	NCAPD3	11	133527547	133599636	1	1.31E-01
GO:51301	85444	LRRCC1	8	86206629	86245567	1	1.33E-01
PAN-PW44	1103	CHAT	10	50487147	50543156	1	1.43E-01
PAN-PW44	6572	SLC18A3	10	50488353	50490772	1	1.43E-01
GO:42493	1103	CHAT	10	50487147	50543156	1	1.43E-01
GO:42493	2567	GABRG3	15	24799263	25451729	2	1.46E-01
GO:42493	64170	CARD9	9	138378229	138387939	1	1.59E-01
GO:51301	1731	SEPT-1	16	30296957	30301672	1	1.80E-01
GO:42493	6609	SMPD1	11	6368231	6372802	1	1.86E-01
GO:42493	1019	CDK4	12	56428270	56432431	1	1.91E-01
GO:51301	1017	CDK2	12	54646826	54652835	1	1.91E-01
GO:51301	1019	CDK4	12	56428270	56432431	1	1.91E-01
PAN-PW44	4640	MYO1A	12	55708568	55730160	1	1.91E-01
GO:42493	2065	ERBB3	12	54760159	54783395	1	1.91E-01
GO:42493	9501	RPH3AL	17	62293	202576	1	1.95E-01
GO:42493	9961	MVP	16	29731591	29766842	5	2.82E-01
GO:51301	29882	ANAPC2	9	139189057	139202878	2	3.95E-01
GO:51301	93426	SYCE1	10	135217395	135232866	1	4.36E-01
GO:42493	1312	COMT	22	18309309	18336530	1	6.33E-01
GO:51301	5413	SEPT-5	22	18081987	18092297	1	6.33E-01

Note: For each gene, the number of case copy number variants hitting that gene is given (if greater than zero), together with gene-specific enrichment p-value.

**Table S2:** Pathway-Specific Enrichment *p* Values in Attention-Deficit/Hyperactivity Disorder (ADHD) and Autism Spectrum Disorder (ASD) Samples for the 100 Pathways Significantly Enriched (*p* < .05) in Both ADHD and ASD

Pathway ID	No. of Genes	*p* (Combined)	*p* (ADHD)	*p* (ADHD No ASD loci)	*p* (ADHD No Case CNV Overlap)	*p* (ASD)	*p* (ASD No ASD Loci)	*p* (ASD No Case CNV Overlap)	Pathway Description
PAN-PW44	89	1.75E-07	3.39E-07	3.06E-07	1.78E-02	5.81E-03	4.70E-03	2.85E-01	Nicotinic acetylcholines receptor signaling pathway
GO:51301	364	7.69E-07	2.19E-04	3.41E-04	1.48E-02	1.64E-03	8.20E-04	4.13E-03	Cell division
GO:42493	322	8.94E-07	4.11E-04	1.67E-04	2.10E-02	5.69E-04	6.92E-04	6.91E-03	Response to drug
GO:5516	147	5.66E-06	2.87E-03	2.51E-03	2.86E-01	9.50E-04	5.97E-04	7.20E-04	Calmodulin binding
GO:5794	1022	9.77E-06	2.10E-03	1.34E-03	7.62E-03	2.35E-03	3.68E-03	3.55E-02	Golgi apparatus
MGI:5620	212	1.15E-05	7.41E-04	6.43E-04	2.14E-02	1.82E-02	1.71E-02	1.00E-01	Abnormal muscle contractility
GO:6195	625	1.80E-05	6.26E-03	5.13E-03	1.28E-01	2.20E-03	1.32E-03	1.46E-03	Purine nucleotide catabolic process
GO:9154	602	1.80E-05	6.26E-03	5.13E-03	1.28E-01	2.20E-03	1.32E-03	1.46E-03	Purine ribonucleotide catabolic process
GO:9261	604	1.80E-05	6.26E-03	5.13E-03	1.28E-01	2.20E-03	1.32E-03	1.46E-03	Ribonucleotide catabolic process
GO:9143	600	1.99E-05	3.75E-03	5.13E-03	1.28E-01	4.24E-03	2.65E-03	3.33E-03	Nucleoside triphosphate catabolic process
GO:9166	642	2.21E-05	9.48E-03	1.24E-02	2.12E-01	2.18E-03	1.31E-03	1.45E-03	Nucleotide catabolic process
GO:9141	640	2.48E-05	2.17E-02	1.26E-02	3.01E-01	9.13E-04	1.35E-03	5.22E-03	Nucleoside triphosphate metabolic process
GO:9144	631	2.50E-05	2.18E-02	1.26E-02	3.01E-01	9.13E-04	1.35E-03	5.22E-03	Purine nucleoside triphosphate metabolic process
GO:9199	628	2.50E-05	2.18E-02	1.26E-02	3.01E-01	9.13E-04	1.35E-03	5.22E-03	Ribonucleoside triphosphate metabolic process
GO:9205	627	2.50E-05	2.18E-02	1.26E-02	3.01E-01	9.13E-04	1.35E-03	5.22E-03	Purine ribonucleoside triphosphate metabolic process
PAN-PW16	69	2.76E-05	1.59E-04	1.45E-04	3.99E-02	2.56E-02	1.81E-02	6.47E-02	Cytoskeletal regulation by Rho GTPase
GO:6633	108	2.77E-05	2.16E-03	2.03E-03	1.52E-01	4.19E-03	2.20E-03	1.25E-03	Fatty acid biosynthetic process
GO:10927	94	2.88E-05	3.29E-03	3.05E-03	2.80E-01	2.48E-03	2.49E-03	8.79E-03	Cellular component assembly involved in morphogenesis
GO:48285	306	2.88E-05	1.59E-02	8.26E-03	3.56E-01	9.87E-04	1.17E-03	3.94E-03	Organelle fission
GO:6461	499	3.01E-05	8.82E-03	9.44E-03	8.83E-02	1.41E-03	1.98E-03	1.68E-02	Protein complex assembly
GO:70271	503	3.01E-05	8.82E-03	9.44E-03	8.83E-02	1.41E-03	1.98E-03	1.68E-02	Protein complex biogenesis
GO:9146	597	3.40E-05	6.26E-03	5.13E-03	1.28E-01	4.24E-03	2.65E-03	3.33E-03	Purine nucleoside triphosphate catabolic process
GO:9203	596	3.40E-05	6.26E-03	5.13E-03	1.28E-01	4.24E-03	2.65E-03	3.33E-03	Ribonucleoside triphosphate catabolic process
GO:9207	596	3.40E-05	6.26E-03	5.13E-03	1.28E-01	4.24E-03	2.65E-03	3.33E-03	Purine ribonucleoside triphosphate catabolic process
GO:16887	362	3.70E-05	9.25E-04	7.28E-04	2.76E-02	2.59E-02	2.35E-02	1.10E-01	ATPase activity
GO:6200	365	3.70E-05	9.25E-04	7.28E-04	2.76E-02	2.59E-02	2.35E-02	1.10E-01	ATP catabolic process
GO:280	293	4.29E-05	5.63E-03	2.31E-03	2.29E-01	4.54E-03	5.86E-03	2.45E-02	Nuclear division
GO:7067	293	4.29E-05	5.63E-03	2.31E-03	2.29E-01	4.54E-03	5.86E-03	2.45E-02	Mitosis
GO:51649	1114	4.91E-05	4.32E-04	4.62E-04	1.76E-03	3.23E-02	4.90E-02	2.68E-01	Establishment of localization in cell
GO:87	301	5.39E-05	1.50E-02	7.86E-03	3.37E-01	2.02E-03	2.50E-03	9.79E-03	M phase of mitotic cell cycle
GO:34655	667	5.55E-05	4.13E-02	5.14E-02	4.00E-01	1.14E-03	6.48E-04	6.08E-04	Nucleobase-containing compound catabolic process
GO:44270	693	5.55E-05	4.13E-02	5.14E-02	4.00E-01	1.14E-03	6.48E-04	6.08E-04	Cellular nitrogen compound catabolic process
GO:6936	171	5.55E-05	3.88E-03	1.18E-03	5.00E-01	5.93E-03	7.17E-03	3.58E-01	Muscle contraction
MGI:4811	331	6.15E-05	1.09E-04	9.37E-05	6.39E-02	3.97E-02	3.21E-02	1.94E-01	Abnormal neuron physiology
MGI:1876	190	6.18E-05	2.29E-02	2.15E-02	2.46E-01	2.09E-03	1.34E-03	3.51E-03	Decreased inflammatory response
GO:8610	397	7.81E-05	2.43E-02	1.64E-02	1.87E-01	1.52E-03	2.13E-03	1.04E-02	Lipid biosynthetic process
GO:72523	631	8.36E-05	3.13E-02	2.70E-02	2.87E-01	2.20E-03	1.32E-03	1.46E-03	Purine-containing compound catabolic process
GO:51656	96	9.17E-05	1.97E-04	4.11E-04	9.02E-02	3.09E-02	2.73E-02	3.83E-01	Establishment of organelle localization
MGI:1756	97	9.69E-05	2.54E-02	2.41E-02	2.75E-01	1.29E-03	1.06E-03	3.93E-03	Abnormal urination
MGI:5278	224	1.01E-04	4.60E-04	4.16E-04	3.21E-02	2.15E-02	2.07E-02	6.82E-02	Abnormal cholesterol homeostasis
GO:16053	234	1.06E-04	3.82E-03	5.86E-03	2.05E-01	1.14E-02	7.04E-03	4.91E-03	Organic acid biosynthetic process
GO:46394	234	1.06E-04	3.82E-03	5.86E-03	2.05E-01	1.14E-02	7.04E-03	4.91E-03	Carboxylic acid biosynthetic process
GO:33559	70	1.15E-04	4.80E-02	4.66E-02	1.24E-02	4.98E-04	2.43E-04	1.35E-04	Unsaturated fatty acid metabolic process
GO:6690	67	1.15E-04	4.80E-02	4.66E-02	1.24E-02	4.98E-04	2.43E-04	1.35E-04	Icosanoid metabolic process
MGI:5294	368	1.15E-04	1.58E-03	1.40E-03	1.50E-01	1.62E-02	1.20E-02	1.14E-01	Abnormal heart ventricle morphology
GO:51641	1302	1.24E-04	1.47E-03	1.65E-03	3.27E-03	3.40E-02	5.11E-02	3.10E-01	Cellular localization
MGI:180	197	1.55E-04	8.00E-04	7.44E-04	5.62E-02	2.11E-02	2.05E-02	8.24E-02	Abnormal circulating cholesterol level
GO:42623	285	1.56E-04	1.87E-03	1.56E-03	5.06E-02	4.77E-02	4.38E-02	2.00E-01	ATPase activity, coupled
GO:279	434	1.95E-04	4.00E-02	2.61E-02	3.92E-01	2.50E-03	3.39E-03	7.70E-03	M phase
GO:6692	30	2.23E-04	4.82E-02	4.68E-02	1.22E-02	1.15E-03	6.55E-04	6.97E-04	Prostanoid metabolic process
GO:46034	385	2.27E-04	1.50E-02	7.89E-03	2.14E-01	1.36E-02	2.10E-02	1.81E-01	ATP metabolic process
MGI:1663	388	2.70E-04	1.31E-02	1.20E-02	9.09E-02	2.03E-02	1.71E-02	3.13E-02	Abnormal digestive system physiology
GO:793	145	2.79E-04	2.41E-03	2.27E-03	1.86E-01	2.62E-02	2.41E-02	6.59E-02	Condensed chromosome
KEGG4020	177	2.94E-04	2.23E-02	2.11E-02	1.26E-01	5.10E-03	3.55E-03	5.72E-02	Calcium signaling pathway
GO:19752	694	3.00E-04	1.51E-02	2.00E-02	1.23E-01	8.88E-03	5.46E-03	6.48E-03	Carboxylic acid metabolic process
GO:42180	709	3.00E-04	1.51E-02	2.00E-02	1.23E-01	8.88E-03	5.46E-03	6.48E-03	Cellular ketone metabolic process
GO:43436	694	3.00E-04	1.51E-02	2.00E-02	1.23E-01	8.88E-03	5.46E-03	6.48E-03	Oxoacid metabolic process
GO:6082	712	3.07E-04	9.49E-03	1.26E-02	7.52E-02	1.36E-02	8.70E-03	1.03E-02	Organic acid metabolic process
GO:43292	135	3.12E-04	3.91E-02	2.08E-02	2.00E-01	2.72E-03	5.56E-04	1.15E-01	Contractile fiber
GO:22857	935	3.45E-04	7.98E-03	5.42E-03	2.25E-01	2.04E-02	2.65E-02	2.66E-01	Transmembrane transporter activity
GO:775	149	3.81E-04	1.97E-03	5.41E-04	9.56E-02	4.10E-02	6.16E-02	3.30E-01	Chromosome, centromeric region
MGI:2462	185	3.85E-04	5.97E-03	8.63E-03	1.68E-01	3.62E-02	5.24E-02	2.56E-01	Abnormal granulocyte physiology
MGI:3947	216	4.01E-04	8.00E-04	7.44E-04	5.62E-02	4.11E-02	3.96E-02	1.33E-01	Abnormal cholesterol level
GO:7613	57	4.13E-04	2.69E-03	2.65E-03	4.74E-01	1.50E-02	1.53E-02	3.53E-01	Memory
GO:33036	1342	4.43E-04	1.75E-02	2.04E-02	9.05E-02	1.38E-02	1.96E-02	1.80E-01	Macromolecule localization
MGI:8873	166	4.48E-04	1.01E-02	8.96E-03	1.26E-01	4.39E-02	4.20E-02	1.40E-01	Increased physiological sensitivity to xenobiotic
GO:5929	195	4.71E-04	1.22E-03	1.09E-03	2.07E-01	4.82E-02	4.42E-02	8.52E-02	Cilium
GO:55074	208	5.40E-04	4.48E-03	4.22E-03	1.93E-02	4.84E-02	3.92E-02	1.81E-01	Calcium ion homeostasis
GO:72507	214	5.40E-04	4.48E-03	4.22E-03	1.93E-02	4.84E-02	3.92E-02	1.81E-01	Divalent inorganic cation homeostasis
MGI:2064	254	5.53E-04	1.06E-02	9.84E-03	4.37E-02	2.28E-02	1.85E-02	1.18E-01	Seizures
GO:44449	121	5.55E-04	3.91E-02	2.08E-02	2.00E-01	5.55E-03	1.30E-03	2.58E-01	Contractile fiber part
MGI:2106	553	6.50E-04	1.72E-02	1.50E-02	1.44E-01	2.86E-02	2.39E-02	4.78E-02	Abnormal muscle physiology
GO:55085	1084	6.77E-04	2.29E-02	1.65E-02	3.51E-01	1.76E-02	2.31E-02	2.30E-01	Transmembrane transport
GO:776	92	6.78E-04	3.18E-03	2.94E-04	4.48E-01	3.30E-02	4.71E-02	3.78E-01	Kinetochore
GO:8104	1100	6.95E-04	3.19E-02	3.69E-02	1.36E-01	1.28E-02	1.76E-02	1.97E-01	Protein localization
GO:43623	172	7.18E-04	4.36E-03	4.08E-03	3.86E-01	2.65E-02	2.11E-02	3.24E-02	Cellular protein complex assembly
GO:71822	596	7.36E-04	1.89E-02	2.09E-02	1.42E-01	1.34E-02	1.85E-02	5.77E-02	Protein complex subunit organization
GO:23061	114	7.63E-04	9.80E-03	7.22E-03	2.32E-03	4.05E-02	5.57E-02	7.95E-02	Signal release
GO:3001	114	7.63E-04	9.80E-03	7.22E-03	2.32E-03	4.05E-02	5.57E-02	7.95E-02	Generation of a signal involved in cell-cell signaling
GO:42470	93	7.94E-04	1.16E-02	1.10E-02	4.27E-01	3.39E-02	2.76E-02	2.33E-01	Melanosome
GO:48770	93	7.94E-04	1.16E-02	1.10E-02	4.27E-01	3.39E-02	2.76E-02	2.33E-01	Pigment granule
GO:5261	282	9.70E-04	2.03E-02	1.34E-02	1.22E-01	1.83E-02	2.69E-02	4.14E-01	Cation channel activity
GO:5694	574	1.12E-03	1.19E-02	7.49E-03	9.19E-02	4.24E-02	6.46E-02	2.88E-01	Chromosome
GO:48878	656	1.20E-03	2.04E-02	2.83E-02	1.61E-02	4.10E-02	3.15E-02	1.53E-01	Chemical homeostasis
GO:42803	484	1.21E-03	8.65E-03	1.18E-02	4.42E-02	4.66E-02	7.05E-02	4.01E-01	Protein homodimerization activity
GO:5215	1194	1.32E-03	2.76E-02	2.09E-02	3.49E-01	3.27E-02	4.32E-02	3.28E-01	Transporter activity
GO:7601	203	1.42E-03	2.04E-02	1.87E-02	2.18E-01	3.00E-02	2.71E-02	3.25E-01	Visual perception
GO:44283	334	2.00E-03	2.33E-02	1.35E-02	4.75E-01	3.33E-02	4.86E-02	8.27E-02	Small molecule biosynthetic process
GO:6631	240	2.10E-03	1.76E-02	1.67E-02	2.95E-01	3.61E-02	2.59E-02	1.75E-02	Fatty acid metabolic process
MGI:6042	414	2.22E-03	2.39E-02	3.39E-02	2.61E-01	3.13E-02	4.46E-02	2.18E-01	Increased apoptosis
GO:44085	1240	2.44E-03	1.42E-02	1.59E-02	6.05E-02	2.00E-02	2.94E-02	4.54E-02	Cellular component biogenesis
GO:32787	344	2.46E-03	1.76E-02	1.67E-02	2.95E-01	2.81E-02	2.07E-02	9.38E-03	Monocarboxylic acid metabolic process
GO:30030	681	2.84E-03	3.37E-02	2.31E-02	2.41E-01	4.19E-02	5.10E-02	1.12E-01	Cell projection organization
GO:42330	530	3.68E-03	4.22E-02	2.99E-02	4.18E-01	4.03E-02	4.99E-02	4.20E-01	Taxis
GO:6935	530	3.68E-03	4.22E-02	2.99E-02	4.18E-01	4.03E-02	4.99E-02	4.20E-01	Chemotaxis
GO:65003	713	3.69E-03	2.93E-02	3.32E-02	1.68E-01	2.23E-02	3.12E-02	4.01E-02	Macromolecular complex assembly
GO:43279	79	4.69E-03	2.31E-02	2.27E-02	4.33E-01	4.21E-02	4.07E-02	3.70E-01	Response to alkaloid
GO:22607	1107	5.06E-03	2.98E-02	3.36E-02	1.36E-01	2.29E-02	3.35E-02	5.65E-02	Cellular component assembly
MGI:3203	78	7.57E-03	4.01E-02	3.95E-02	4.17E-01	4.45E-02	4.16E-02	2.53E-01	Increased neuron apoptosis
MGI:1402	231	7.81E-03	2.22E-02	2.18E-02	3.97E-01	4.31E-02	3.33E-02	1.52E-01	Hypoactivity

Note: Enrichment p-values are also provided when known ASD loci and overlapping case copy number variants between disorders have been removed. CNV: copy number variant

**Table S3:** Pathway-Specific Enrichment *p* Values in Attention-Deficit/Hyperactivity Disorder (ADHD) and Autism Spectrum Disorder (ASD) Samples for the 100 Pathways Significantly Enriched (*p* < .05) in Both ADHD and ASD When Deletions and Duplications Are Analyzed Separately

Pathway	*p* (Combined)	*p* (ADHD)	*p* (ADHD del)	*p* (ADHD dup)	*p* (ASD)	*p* (ASD del)	*p* (ASD dup)	Function
PAN-PW44	1.75E-07	3.39E-07	3.84E-01	1.41E-07	5.81E-03	3.49E-03	6.72E-02	Nicotinic acetylcholine receptor signaling pathway
GO:51301	7.69E-07	2.19E-04	3.81E-01	7.30E-05	1.64E-03	2.54E-01	2.95E-03	Cell division
GO:42493	8.94E-07	4.11E-04	4.01E-01	1.18E-04	5.69E-04	8.78E-04	3.24E-02	Response to drug
GO:5516	5.66E-06	2.87E-03	N/A	2.87E-03	9.50E-04	7.33E-02	4.65E-03	Calmodulin binding
GO:5794	9.77E-06	2.10E-03	2.40E-01	1.15E-03	2.35E-03	1.02E-02	2.62E-02	Golgi apparatus
MGI:5620	1.15E-05	7.41E-04	2.24E-01	1.80E-03	1.82E-02	1.22E-01	3.64E-02	Abnormal muscle contractility
GO:6195	1.80E-05	6.26E-03	4.10E-02	5.83E-03	2.20E-03	1.68E-02	3.29E-02	Purine nucleotide catabolic process
GO:9154	1.80E-05	6.26E-03	4.10E-02	5.83E-03	2.20E-03	1.68E-02	3.29E-02	Purine ribonucleotide catabolic process
GO:9261	1.80E-05	6.26E-03	4.10E-02	5.83E-03	2.20E-03	1.68E-02	3.29E-02	Ribonucleotide catabolic process
GO:9143	1.99E-05	3.75E-03	4.08E-02	5.83E-03	4.24E-03	4.90E-02	3.30E-02	Nucleoside triphosphate catabolic process
GO:9166	2.21E-05	9.48E-03	4.08E-02	1.45E-02	2.18E-03	1.68E-02	3.29E-02	Nucleotide catabolic process
GO:9141	2.48E-05	2.17E-02	3.82E-01	1.41E-02	9.13E-04	3.63E-03	3.07E-02	Nucleoside triphosphate metabolic process
GO:9144	2.50E-05	2.18E-02	3.82E-01	1.41E-02	9.13E-04	3.63E-03	3.07E-02	Purine nucleoside triphosphate metabolic process
GO:9199	2.50E-05	2.18E-02	3.82E-01	1.41E-02	9.13E-04	3.63E-03	3.07E-02	Ribonucleoside triphosphate metabolic process
GO:9205	2.50E-05	2.18E-02	3.82E-01	1.41E-02	9.13E-04	3.63E-03	3.07E-02	Purine ribonucleoside triphosphate metabolic process
PAN-PW16	2.76E-05	1.59E-04	N/A	1.59E-04	2.56E-02	7.33E-02	9.99E-02	Cytoskeletal regulation by Rho GTPase
GO:6633	2.77E-05	2.16E-03	2.69E-01	1.58E-03	4.19E-03	7.33E-02	2.12E-02	Fatty acid biosynthetic process
GO:10927	2.88E-05	3.29E-03	4.81E-01	3.04E-03	2.48E-03	4.87E-02	1.60E-02	Cellular component assembly involved in morphogenesis
GO:48285	2.88E-05	1.59E-02	2.06E-01	1.96E-02	9.87E-04	7.52E-04	7.22E-02	Organelle fission
GO:6461	3.01E-05	8.82E-03	2.65E-01	9.72E-03	1.41E-03	1.27E-02	2.41E-02	Protein complex assembly
GO:70271	3.01E-05	8.82E-03	2.65E-01	9.72E-03	1.41E-03	1.27E-02	2.41E-02	Protein complex biogenesis
GO:9146	3.40E-05	6.26E-03	4.10E-02	5.83E-03	4.24E-03	4.90E-02	3.30E-02	Purine nucleoside triphosphate catabolic process
GO:9203	3.40E-05	6.26E-03	4.10E-02	5.83E-03	4.24E-03	4.90E-02	3.30E-02	Ribonucleoside triphosphate catabolic process
GO:9207	3.40E-05	6.26E-03	4.10E-02	5.83E-03	4.24E-03	4.90E-02	3.30E-02	Purine ribonucleoside triphosphate catabolic process
GO:6200	3.70E-05	9.25E-04	4.10E-02	1.73E-03	2.59E-02	4.90E-02	1.24E-01	ATP catabolic process
GO:16887	3.70E-05	9.25E-04	4.10E-02	1.73E-03	2.59E-02	4.90E-02	1.24E-01	ATPase activity
GO:280	4.29E-05	5.63E-03	2.06E-01	7.10E-03	4.54E-03	9.60E-03	7.22E-02	Nuclear division
GO:7067	4.29E-05	5.63E-03	2.06E-01	7.10E-03	4.54E-03	9.60E-03	7.22E-02	Mitosis
GO:51649	4.91E-05	4.32E-04	7.35E-02	1.51E-03	3.23E-02	2.53E-02	1.17E-01	Establishment of localization in cell
GO:87	5.39E-05	1.50E-02	4.13E-01	7.10E-03	2.02E-03	9.60E-03	3.43E-02	M phase of mitotic cell cycle
GO:6936	5.55E-05	3.88E-03	4.70E-01	2.05E-03	5.93E-03	1.95E-03	6.60E-02	Muscle contraction
GO:34655	5.55E-05	4.13E-02	4.46E-01	3.20E-02	1.14E-03	1.68E-02	1.94E-02	Nucleobase-containing compound catabolic process
GO:44270	5.55E-05	4.13E-02	4.46E-01	3.20E-02	1.14E-03	1.68E-02	1.94E-02	Cellular nitrogen compound catabolic process
MGI:4811	6.15E-05	1.09E-04	4.28E-01	2.17E-05	3.97E-02	1.02E-01	1.36E-01	Abnormal neuron physiology
MGI:1876	6.18E-05	2.29E-02	4.27E-01	2.48E-02	2.09E-03	7.33E-02	1.06E-02	Decreased inflammatory response
GO:8610	7.81E-05	2.43E-02	2.03E-01	3.79E-02	1.52E-03	1.43E-04	6.63E-02	Lipid biosynthetic process
GO:72523	8.36E-05	3.13E-02	4.48E-01	1.48E-02	2.20E-03	1.68E-02	3.29E-02	Purine-containing compound catabolic process
GO:51656	9.17E-05	1.97E-04	N/A	1.97E-04	3.09E-02	7.33E-02	8.30E-02	Establishment of organelle localization
MGI:1756	9.69E-05	2.54E-02	4.81E-01	2.43E-02	1.29E-03	1.82E-02	1.16E-02	Abnormal urination
MGI:5278	1.01E-04	4.60E-04	4.81E-01	1.70E-04	2.15E-02	1.39E-03	2.41E-01	Abnormal cholesterol homeostasis
GO:16053	1.06E-04	3.82E-03	2.67E-01	4.33E-03	1.14E-02	1.89E-02	8.50E-02	Organic acid biosynthetic process
GO:46394	1.06E-04	3.82E-03	2.67E-01	4.33E-03	1.14E-02	1.89E-02	8.50E-02	Carboxylic acid biosynthetic process
GO:6690	1.15E-04	4.80E-02	N/A	4.80E-02	4.98E-04	7.33E-02	3.14E-03	Icosanoid metabolic process
GO:33559	1.15E-04	4.80E-02	N/A	4.80E-02	4.98E-04	7.33E-02	3.14E-03	Unsaturated fatty acid metabolic process
MGI:5294	1.15E-04	1.58E-03	N/A	1.58E-03	1.62E-02	1.21E-01	4.83E-02	Abnormal heart ventricle morphology
GO:51641	1.24E-04	1.47E-03	3.43E-01	2.03E-03	3.40E-02	5.81E-02	9.01E-02	Cellular localization
MGI:180	1.55E-04	8.00E-04	4.81E-01	3.43E-04	2.11E-02	4.89E-03	1.57E-01	Abnormal circulating cholesterol level
GO:42623	1.56E-04	1.87E-03	1.04E-01	1.73E-03	4.77E-02	1.38E-01	1.25E-01	ATPase activity, coupled
GO:279	1.95E-04	4.00E-02	2.42E-01	1.02E-02	2.50E-03	7.81E-02	9.15E-03	M phase
GO:6692	2.23E-04	4.82E-02	N/A	4.82E-02	1.15E-03	7.33E-02	6.28E-03	Prostanoid metabolic process
GO:46034	2.27E-04	1.50E-02	3.82E-01	1.19E-02	1.36E-02	3.63E-03	1.62E-01	ATP metabolic process
MGI:1663	2.70E-04	1.31E-02	6.54E-02	3.97E-02	2.03E-02	1.82E-02	9.62E-02	Abnormal digestive system physiology
GO:793	2.79E-04	2.41E-03	N/A	2.41E-03	2.62E-02	2.54E-01	1.60E-02	Condensed chromosome
KEGG4020	2.94E-04	2.23E-02	2.59E-01	9.52E-03	5.10E-03	1.33E-01	2.12E-02	Calcium signaling pathway
GO:19752	3.00E-04	1.51E-02	4.14E-01	1.23E-02	8.88E-03	3.86E-01	1.34E-02	Carboxylic acid metabolic process
GO:42180	3.00E-04	1.51E-02	4.14E-01	1.23E-02	8.88E-03	3.86E-01	1.34E-02	Cellular ketone metabolic process
GO:43436	3.00E-04	1.51E-02	4.14E-01	1.23E-02	8.88E-03	3.86E-01	1.34E-02	Oxoacid metabolic process
GO:6082	3.07E-04	9.49E-03	4.14E-01	7.07E-03	1.36E-02	3.86E-01	2.10E-02	Organic acid metabolic process
GO:43292	3.12E-04	3.91E-02	4.62E-01	5.44E-02	2.72E-03	1.95E-03	4.78E-02	Contractile fiber
GO:22857	3.45E-04	7.98E-03	4.05E-01	1.25E-02	2.04E-02	3.58E-03	2.71E-01	Transmembrane transporter activity
GO:775	3.81E-04	1.97E-03	4.70E-01	1.53E-03	4.10E-02	2.99E-02	1.72E-01	Chromosome, centromeric region
MGI:2462	3.85E-04	5.97E-03	4.26E-01	1.32E-02	3.62E-02	1.95E-03	3.05E-01	Abnormal granulocyte physiology
MGI:3947	4.01E-04	8.00E-04	4.81E-01	3.43E-04	4.11E-02	4.89E-03	2.41E-01	Abnormal cholesterol level
GO:7613	4.13E-04	2.69E-03	1.40E-01	1.78E-04	1.50E-02	5.06E-05	3.72E-01	Memory
GO:33036	4.43E-04	1.75E-02	7.26E-02	3.23E-02	1.38E-02	4.77E-02	9.49E-02	Macromolecule localization
MGI:8873	4.48E-04	1.01E-02	6.74E-02	3.12E-02	4.39E-02	4.82E-01	2.12E-02	Increased physiological sensitivity to xenobiotic
GO:5929	4.71E-04	1.22E-03	1.08E-01	1.48E-03	4.82E-02	5.36E-03	2.70E-01	Cilium
GO:55074	5.40E-04	4.48E-03	1.76E-01	8.83E-03	4.84E-02	4.35E-03	3.13E-01	Calcium ion homeostasis
GO:72507	5.40E-04	4.48E-03	1.76E-01	8.83E-03	4.84E-02	4.35E-03	3.13E-01	Divalent inorganic cation homeostasis
MGI:2064	5.53E-04	1.06E-02	2.68E-01	2.55E-02	2.28E-02	3.89E-03	1.70E-01	Seizures
GO:44449	5.55E-04	3.91E-02	4.62E-01	5.44E-02	5.55E-03	1.95E-03	7.75E-02	Contractile fiber part
MGI:2106	6.50E-04	1.72E-02	2.19E-01	1.88E-03	2.86E-02	1.64E-02	1.99E-01	Abnormal muscle physiology
GO:55085	6.77E-04	2.29E-02	3.71E-01	2.17E-02	1.76E-02	3.58E-03	2.42E-01	Transmembrane transport
GO:776	6.78E-04	3.18E-03	4.70E-01	2.88E-03	3.30E-02	2.99E-02	1.25E-01	Kinetochore
GO:8104	6.95E-04	3.19E-02	7.27E-02	7.06E-02	1.28E-02	5.43E-02	7.38E-02	Protein localization
GO:43623	7.18E-04	4.36E-03	N/A	4.36E-03	2.65E-02	4.48E-02	1.39E-01	Cellular protein complex assembly
GO:71822	7.36E-04	1.89E-02	2.65E-01	2.07E-02	1.34E-02	1.27E-02	1.51E-01	Protein complex subunit organization
GO:3001	7.63E-04	9.80E-03	1.59E-01	2.36E-02	4.05E-02	1.85E-03	2.83E-01	Generation of a signal involved in cell-cell signaling
GO:23061	7.63E-04	9.80E-03	1.59E-01	2.36E-02	4.05E-02	1.85E-03	2.83E-01	Signal release
GO:42470	7.94E-04	1.16E-02	4.81E-01	8.16E-03	3.39E-02	7.33E-02	9.52E-02	Melanosome
GO:48770	7.94E-04	1.16E-02	4.81E-01	8.16E-03	3.39E-02	7.33E-02	9.52E-02	Pigment granule
GO:5261	9.70E-04	2.03E-02	4.18E-01	3.04E-02	1.83E-02	1.30E-05	3.78E-01	Cation channel activity
GO:5694	1.12E-03	1.19E-02	4.73E-01	6.90E-03	4.24E-02	6.98E-02	1.66E-01	Chromosome
GO:48878	1.20E-03	2.04E-02	3.38E-01	1.69E-02	4.10E-02	1.88E-02	2.57E-01	Chemical homeostasis
GO:42803	1.21E-03	8.65E-03	1.08E-01	1.06E-03	4.66E-02	7.80E-04	4.00E-01	Protein homodimerization activity
GO:5215	1.32E-03	2.76E-02	3.73E-01	3.46E-02	3.27E-02	8.29E-03	3.08E-01	Transporter activity
GO:7601	1.42E-03	2.04E-02	3.02E-01	2.37E-02	3.00E-02	3.49E-03	2.12E-01	Visual perception
GO:44283	2.00E-03	2.33E-02	2.66E-01	1.90E-02	3.33E-02	6.84E-03	2.65E-01	Small molecule biosynthetic process
GO:6631	2.10E-03	1.76E-02	2.69E-01	1.26E-02	3.61E-02	3.23E-01	6.73E-02	Fatty acid metabolic process
MGI:6042	2.22E-03	2.39E-02	3.33E-01	2.60E-02	3.13E-02	1.45E-04	4.90E-01	Increased apoptosis
GO:44085	2.44E-03	1.42E-02	4.24E-01	1.76E-02	2.00E-02	2.83E-02	1.33E-01	Cellular component biogenesis
GO:32787	2.46E-03	1.76E-02	2.69E-01	1.26E-02	2.81E-02	1.23E-01	8.62E-02	Monocarboxylic acid metabolic process
GO:30030	2.84E-03	3.37E-02	2.89E-01	4.46E-02	4.19E-02	4.34E-03	3.15E-01	Cell projection organization
GO:6935	3.68E-03	4.22E-02	1.11E-01	4.87E-02	4.03E-02	3.70E-03	2.93E-01	Chemotaxis
GO:42330	3.68E-03	4.22E-02	1.11E-01	4.87E-02	4.03E-02	3.70E-03	2.93E-01	Taxis
GO:65003	3.69E-03	2.93E-02	2.65E-01	2.64E-02	2.23E-02	1.02E-01	6.84E-02	Macromolecular complex assembly
GO:43279	4.69E-03	2.31E-02	1.85E-01	7.25E-03	4.21E-02	1.55E-02	1.70E-01	Response to alkaloid
GO:22607	5.06E-03	2.98E-02	4.86E-01	2.71E-02	2.29E-02	2.82E-02	1.48E-01	Cellular component assembly
MGI:3203	7.57E-03	4.01E-02	4.31E-01	1.58E-02	4.45E-02	4.01E-03	2.41E-01	Increased neuron apoptosis
MGI:1402	7.81E-03	2.22E-02	1.83E-01	2.45E-03	4.31E-02	1.25E-01	1.69E-01	Hypoactivity

Note: del: deletion; dup: duplication

**Table S4:** Complete List of Case Copy Number Variants (CNVs) >500 kb (Attention-Deficit/Hyperactivity Disorder [ADHD] and Autism Spectrum Disorder [ASD]) Used in the Analyses

Case (ADHD/ ASD)	Chr	Start (bp)	End (bp)	Del/Dup	Inherited?	Overlap Autism Region?	Overlap CNVs of Other Disorder?	Contribution to Top 20 Pathways	Genes in Top 3 Pathways	All Genes Hit by CNV
ASD	1	2678023	3198235	Dup	Inherited	No	No			ACTRT2, PRDM16,
ASD	1	30334653	30951250	Del	Inherited	No	No			
ASD	1	32696367	33315639	Dup	Inherited	No	No	GO:9154, GO:9205, GO:6195, GO:9143, GO:9144, GO:9261, GO:9199, GO:9166, GO:9141, MGI:5620,		ZBTB8B, ZBTB8A, ZBTB8OS, RBBP4, SYNC, KIAA1522, YARS, S100PBP, FNDC5, HPCA, TMEM54, RNF19B, AK2,
ASD	1	57997961	58568421	Dup	Inherited	No	No			DAB1,
ASD	1	112992330	113553163	Dup	Inherited	No	No	GO:6461, GO:42493, GO:9154, GO:9205, GO:6195, GO:9143, GO:9144, GO:9261, GO:9199, GO:9166, GO:9141, GO:51301, PAN-PW:16,	RHOC,	CAPZA1, MOV10, RHOC, PPM1J, FAM19A3, LOC128322, SLC16A1, LRIG2,
ASD	1	144099494	144627859	Del	Inherited	No	No	GO:42493, MGI:5620, GO:48285, GO:5794,	TXNIP, PDZK1,	HFE2, TXNIP, POLR3GL, ANKRD34A, LIX1L, RBM8A, PEX11B, ITGA10, ANKRD35, PIAS3, NUDT17, POLR3C, RNF115, CD160, PDZK1, GPR89A, GPR89C,
ASD	1	144838594	146308287	Dup	Inherited	No	Yes	GO:9154, GO:9205, GO:6195, GO:9143, GO:9144, GO:9261, GO:9199, GO:9166, GO:9141, GO:5794,		PRKAB2, FMO5, CHD1L, BCL9, ACP6, GJA5, GJA8, GPR89B, NBPF24,
ASD	1	155976899	156525831	Dup	Inherited	No	No			FCRL2, FCRL1, CD5L, KIRREL, CD1D, CD1A,
ASD	1	240371271	241192975	Del	Inherited	No	No			PLD5,
ASD	1	244036261	245191978	Del	Inherited	No	No	GO:5794, GO:48285, GO:51301, GO:6461,	AHCTF1 ,	SMYD3, TFB2M, CNST, SCCPDH, LOC100130097, AHCTF1,
ASD	2	20961443	21478579	Dup	Inherited	No	No			APOB, LOC100129278,
ASD	2	32480121	33184723	Dup	Inherited	No	Yes			BIRC6, TTC27, LTBP1,
ASD	2	32480121	33174461	Dup	Inherited	No	Yes			BIRC6, TTC27, LTBP1,
ASD	2	32483938	33181898	Dup	Inherited	No	Yes			BIRC6, TTC27, LTBP1,
ASD	2	89685181	90970848	Del	Inherited	No	No			IGK@,
ASD	2	110050606	110615080	Dup	Inherited	No	No	GO:5794,		MALL, NPHP1,
ASD	2	131196881	131747151	Dup	Inherited	No	No			GPR148, AMER3, ARHGEF4, FAM168B, PLEKHB2, POTEE,
ASD	2	171063444	171826719	Dup	Inherited	No	No	PAN-PW:44, GO:42493, GO:5794,	MYO3B, GAD1,	MYO3B, SP5, ERICH2, GAD1, GORASP2, TLK1,
ASD	2	182777089	184116208	Dup	Inherited	No	No	GO:5516,		PDE1A, DNAJC10, FRZB, NCKAP1, DUSP19, NUP35,
ASD	3	227344	1486108	Dup	Inherited	No	Yes			CHL1, CNTN6,
ASD	3	464181	1251877	Dup	Inherited	No	Yes			CNTN6,
ASD	3	2365612	2875100	Del	Inherited	Yes	No			CNTN4,
ASD	3	38475193	39565199	Dup	Inherited	No	No	MGI:5620, GO:42493, GO:5794, GO:48285, GO:10927,	SCN11A ,	ACVR2B, EXOG, SCN5A, SCN10A, SCN11A, WDR48, GORASP1, TTC21A, CSRNP1, XIRP1, DSTNP4, CX3CR1, CCR8, SLC25A38, RPSA, MOBP,
ASD	3	73185165	73722969	Dup	Inherited	No	No			PPP4R2, EBLN2, PDZRN3,
ASD	3	97474308	98204406	Dup	Inherited	No	Yes			EPHA6,
ASD	3	100079301	100925499	Dup	Inherited	No	No			DCBLD2, COL8A1,
ASD	3	112120161	112961027	Del	Inherited	No	No			PVRL3, CD96, ZBED2, PLCXD2,
ASD	3	165273764	165817130	Del	Inherited	No	No			
ASD	4	34802932	35676439	Dup	Inherited	No	No			
ASD	4	76098820	76815811	Dup	Inherited	No	Yes	GO:5794,		PARM1, RCHY1, THAP6, C4orf26, CDKL2, G3BP2,
ASD	4	115072154	115794717	Dup	Inherited	No	No			ARSJ, UGT8,
ASD	4	162009237	162934619	Dup	Inherited	No	No			FSTL5,
ASD	4	185835398	186412560	Dup	Inherited	No	No	GO:48285,		CCDC111, MLF1IP, ACSL1, LOC100129240, HELT, SLC25A4, KIAA1430, SNX25,
ASD	4	188554375	189071169	Dup	Inherited	No	No			
ASD	4	188698152	189493792	Dup	Inherited	No	No			ZFP42, TRIML2, TRIML1,
ASD	4	189533070	190718765	Del	Inherited	No	Yes			
ASD	5	12454130	12960694	Del	Inherited	No	No			
ASD	5	19532212	20357961	Del	Inherited	No	No			CDH18,
ASD	5	25659711	26168705	Del	Inherited	No	No			
ASD	5	89477076	90142864	Dup	Inherited	No	No	GO:51301, GO:48285,	CETN3,	CETN3, MBLAC2, POLR3G, LYSMD3, GPR98,
ASD	5	104047191	104600670	Del	Inherited	No	No			
ASD	5	104486852	105058907	Dup	Inherited	No	No			
ASD	5	110263975	110789999	Dup	Inherited	No	No	GO:5516,		TSLP, WDR36, CAMK4,
ASD	5	125084106	126044143	Dup	Inherited	No	No			GRAMD3, ALDH7A1, PHAX, C5orf48,
ASD	6	7076523	7622856	Dup	Inherited	No	No			RREB1, SSR1, CAGE1, RIOK1, LOC644058, DSP, SNRNP48,
ASD	6	57336322	58188685	Dup	Inherited	No	No			PRIM2,
ASD	7	47939559	48969654	Del	Inherited	No	No	GO:5794, GO:9166, GO:9154, GO:9205, GO:6195, GO:9143, GO:9144, GO:9261, GO:9199, GO:9141,		PKD1L1, HUS1, 03, C7orf57, UPP1, ABCA13,
ASD	7	61649656	62372905	Dup	Inherited	No	No			
ASD	7	61667556	62336389	Dup	Inherited	No	No			
ASD	7	61681059	62336389	Dup	Inherited	No	No			
ASD	7	61681059	62336389	Dup	Inherited	No	No			
ASD	7	87986401	89721065	Dup	Inherited	No	No	GO:5794,		ZNF804B, C7orf62, STEAP1, STEAP2, C7orf63,
ASD	7	88690424	89260572	Dup	Inherited	No	No			ZNF804B,
ASD	7	88697197	89260572	Dup	Inherited	No	No			ZNF804B,
ASD	7	108956186	109951683	Dup	Inherited	No	No			
ASD	7	142981711	143506472	Dup	Inherited	No	No			FAM115C, CTAGE6P, FAM115A, OR2F2, OR2F1, OR6B1, OR2A5, OR2A25, OR2A12, OR2A2, OR2A14,
ASD	8	2334306	3290879	Dup	Inherited	No	No			CSMD1,
ASD	8	18617115	19445855	Dup	Inherited	No	Yes	GO:5794,		PSD3, SH2D4A, CSGALNACT1,
ASD	8	18774097	19556817	Dup	Inherited	No	Yes	GO:5794,		PSD3, SH2D4A, CSGALNACT1,
ASD	8	89476937	90298110	Dup	Inherited	No	Yes			
ASD	8	124683548	125559395	Del	Inherited	No	No			KLHL38, ANXA13, FAM91A1, FER1L6, TMEM65, TRMT12, RNF139,
ASD	9	92645	733353	Dup	Inherited	No	No			FOXD4, CBWD1, C9orf66, DOCK8, KANK1,
ASD	9	29908652	30428383	Del	Inherited	No	No			
ASD	9	29908652	30428383	Del	Inherited	No	No			
ASD	9	72482886	73063512	Del	Inherited	No	No			TRPM3,
ASD	10	42600836	43271395	Dup	Inherited	No	No	GO:9154, GO:9205, GO:6195, GO:9143, GO:9144, GO:9261, GO:9199, GO:9166, GO:9141, GO:5794,		BMS1, RET, CSGALNACT2, RASGEF1A, FXYD4, HNRNPF,
ASD	10	44956873	45478525	Dup	Inherited	No	No	GO:6633,		OR13A1, ALOX5, MARCH-8, ZFAND4,
ASD	10	134676107	135272450	Dup	Inherited	No	No	GO:6461, GO:51301,	SYCE1,	GPR123, KNDC1, UTF1, VENTX, ADAM8, LOC100128697, TUBGCP2, ZNF511, CALY, PRAP1, FUOM, ECHS1, PAOX, MTG1, SPRN, CYP2E1, SYCE1,
ASD	11	9978367	10670008	Del	Inherited	No	No	GO:6461, MGI:5620, GO:9154, GO:6195, GO:9261, GO:9166,		SBF2, ADM, AMPD3, RNF141, LYVE1, MRVI1,
ASD	11	48332180	48914233	Del	Inherited	No	No			OR4A47,
ASD	11	89389893	91075549	Dup	Inherited	No	No			UBTFL1, NAALAD2, CHORDC1,
ASD	12	122630871	125627131	Dup	Inherited	No	No	GO:5794, GO:6461, GO:9154, GO:9205, GO:6195, GO:9143, GO:9144, GO:9261, GO:9199, GO:9166, GO:9141, GO:10927, GO:42493,	AACS,	TMED2, DDX55, EIF2B1, GTF2H3, TCTN2, ATP6V0A2, DNAH10, CCDC92, ZNF664, FAM101A, NCOR2, SCARB1, UBC, DHX37, BRI3BP, AACS, LOC100129380, TMEM132B,
ASD	14	26685418	28245016	Dup	Inherited	No	No			
ASD	15	20303106	20836955	Del	Inherited	No	No			TUBGCP5, CYFIP1, NIPA2, NIPA1,
ASD	15	20303106	20807351	Dup	Inherited	No	No			TUBGCP5, CYFIP1, NIPA2, NIPA1,
ASD	15	26762141	30436163	Del	Inherited	No	Yes	GO:5794, PAN-PW:44,	CHRNA7 ,	APBA2, NDNL2, TJP1, GOLGA8J, CHRFAM7A, GOLGA8R, GOLGA8H, ARHGAP11B, FAN1, MTMR10, TRPM1, LOC283710, KLF13, OTUD7A, LOC100130857, CHRNA7,
ASD	15	26887815	28157206	Dup	Inherited	No	Yes	GO:5794,		APBA2, NDNL2, TJP1,
ASD	15	28705540	30436163	Del	Inherited	No	Yes	PAN-PW:44,	CHRNA7 ,	ARHGAP11B, FAN1, MTMR10, TRPM1, LOC283710, KLF13, OTUD7A, LOC100130857, CHRNA7,
ASD	15	29762552	30302973	Dup	Inherited	No	Yes	PAN-PW:44,	CHRNA7 ,	LOC100130857, CHRNA7,
ASD	15	29780769	30303265	Dup	Inherited	No	Yes	PAN-PW:44,	CHRNA7 ,	LOC100130857, CHRNA7,
ASD	15	29792536	30303265	Dup	Inherited	No	Yes	PAN-PW:44,	CHRNA7 ,	LOC100130857, CHRNA7,
ASD	15	34016557	34695083	Del	Inherited	No	No			
ASD	16	5312482	5968452	Dup	Inherited	No	No			
ASD	16	7787398	8567137	Dup	Inherited	No	No			TMEM114,
ASD	16	14771033	16307313	Del	Inherited	No	Yes	GO:51301, GO:48285, GO:5516, GO:10927, GO:6461, PAN-PW:16, PAN-PW:44, MGI:5620, GO:5794, GO:42493, GO:9154, GO:9205, GO:6195, GO:9143, GO:9144, GO:9261, GO:9199, GO:9166, GO:9141, GO:6633,	NDE1, MYH11, ABCC1, ABCC6,	NOMO1, NPIP, PDXDC1, NTAN1, RRN3, MPV17L, C16orf45, KIAA0430, NDE1, MYH11, FOPNL, ABCC1, ABCC6, NOMO3,
ASD	16	15387380	16256106	Dup	Inherited	No	Yes	GO:51301, GO:48285, GO:5516, GO:10927, GO:6461, PAN-PW:16, PAN-PW:44, MGI:5620, GO:5794, GO:42493, GO:9154, GO:9205, GO:6195, GO:9143, GO:9144, GO:9261, GO:9199, GO:9166, GO:9141, GO:6633,	NDE1, MYH11, ABCC1, ABCC6,	MPV17L, C16orf45, KIAA0430, NDE1, MYH11, FOPNL, ABCC1, ABCC6, NOMO3,
ASD	16	15387380	16270740	Dup	Inherited	No	Yes	GO:51301, GO:48285, GO:5516, GO:10927, GO:6461, PAN-PW:16, PAN-PW:44, MGI:5620, GO:5794, GO:42493, GO:9154, GO:9205, GO:6195, GO:9143, GO:9144, GO:9261, GO:9199, GO:9166, GO:9141, GO:6633,	NDE1, MYH11, ABCC1, ABCC6,	MPV17L, C16orf45, KIAA0430, NDE1, MYH11, FOPNL, ABCC1, ABCC6, NOMO3,
ASD	16	21788416	22351124	Del	Inherited	No	Yes	GO:5516,		61E3.4, UQCRC2, PDZD9, C16orf52, VWA3A, EEF2K, POLR3E, CDR2,
ASD	16	29502984	30127026	Dup	Inherited	Yes	Yes	GO:6461, GO:5794, GO:48285, GO:42493, GO:9205, GO:9144, GO:9199, GO:9141,	MVP,	SPN, QPRT, C16orf54, ZG16, KIF22, MAZ, PRRT2, MVP, PAGR1, CDIPT, SEZ6L2, ASPHD1, KCTD13, TMEM219, TAOK2, HIRIP3, INO80E, DOC2A, C16orf92, FAM57B, ALDOA, PPP4C, TBX6, YPEL3, GDPD3, MAPK3, CORO1A, BOLA2B, SLX1A, SULT1A3,
ASD	16	60027157	61668976	Del	Inherited	No	No			CDH8,
ASD	16	80739109	82225301	Dup	Inherited	No	No			MPHOSPH6, CDH13,
ASD	16	82750490	83386383	Dup	Inherited	No	No	GO:6461, GO:42493, GO:5794, GO:9154, GO:9205, GO:6195, GO:9143, GO:9144, GO:9261, GO:9199, GO:9166, GO:9141,	WFDC1,	DNAAF1, TAF1C, ADAD2, KCNG4, WFDC1, ATP2C2, KIAA1609, COTL1, KLHL36, USP10,
ASD	17	2852848	3536216	Dup	Inherited	No	No	GO:5516, GO:9205, GO:9144, GO:9199, GO:9141,		RAP1GAP2, OR1D5, OR1D2, OR1G1, OR1A2, OR1A1, OR3A2, OR3A1, OR1E1, OR3A3, OR1E2, SPATA22, ASPA, TRPV3, TRPV1, SHPK, CTNS, TAX1BP3, EMC6, P2RX5,
ASD	17	14040467	15423806	Del	Inherited	No	No	GO:48285, GO:6461, GO:5794, GO:10927,		COX10, CDRT15, HS3ST3B1, PMP22, TEKT3, CDRT4, TVP23C,
ASD	17	48424668	49111866	Dup	Inherited	No	No			
ASD	17	69345596	70202513	Dup	Inherited	No	No	GO:10927,		RPL38, TTYH2, DNAI2, KIF19, BTBD17, GPR142, GPRC5C, CD300A, CD300LB, CD300C, LOC100130520, CD300LD, C17orf77, CD300E, RAB37, CD300LF,
ASD	18	10561904	11907410	Dup	Inherited	No	No	GO:9154, GO:9205, GO:6195, GO:9143, GO:9144, GO:9261, GO:9199, GO:9166, GO:9141, GO:51301, GO:5794,	CHMP1B ,	PIEZO2, SLC35G4, GNAL, CHMP1B, MPPE1,
ASD	18	11203354	12065555	Dup	Inherited	No	No	GO:9154, GO:9205, GO:6195, GO:9143, GO:9144, GO:9261, GO:9199, GO:9166, GO:9141, GO:51301, GO:5794,	CHMP1B ,	SLC35G4, GNAL, CHMP1B, MPPE1, IMPA2,
ASD	18	66234261	66907023	Dup	Inherited	No	No			
ASD	18	69788042	70538070	Dup	Inherited	No	No			FBXO15, TIMM21, CYB5A, C18orf63, FAM69C, CNDP2, CNDP1, ZNF407,
ASD	19	32455280	32988248	Dup	Inherited	No	Yes			
ASD	20	1761373	2500984	Dup	Inherited	No	No	GO:9154, GO:9205, GO:6195, GO:9143, GO:9144, GO:9261, GO:9199, GO:9166, GO:9141, GO:6461,		SIRPA, PDYN, STK35, TGM3, TGM6, SNRPB, ZNF343, TMC2,
ASD	20	5250653	5767545	Dup	Inherited	No	No			GPCPD1, C20orf196,
ASD	22	19051464	19795780	Dup	Inherited	No	Yes	GO:51301, GO:5794, PAN-PW:44, GO:6461,	KLHL22 , SNAP29 ,	USP41, ZNF74, SCARF2, KLHL22, MED15, PI4KA, SERPIND1, SNAP29, CRKL, AIFM3, LZTR1, THAP7, P2RX6, SLC7A4,
ASD	22	19051464	19793730	Dup	Inherited	No	Yes	GO:51301, GO:5794, PAN-PW:44, GO:6461,	KLHL22 , SNAP29 ,	USP41, ZNF74, SCARF2, KLHL22, MED15, PI4KA, SERPIND1, SNAP29, CRKL, AIFM3, LZTR1, THAP7, P2RX6, SLC7A4,
ASD	22	19063495	19793730	Dup	Inherited	No	Yes	GO:51301, GO:5794, PAN-PW:44, GO:6461,	KLHL22 , SNAP29 ,	ZNF74, SCARF2, KLHL22, MED15, PI4KA, SERPIND1, SNAP29, CRKL, AIFM3, LZTR1, THAP7, P2RX6, SLC7A4,
ASD	22	21310351	21984436	Dup	Inherited	No	No	GO:6633, GO:9154, GO:9205, GO:6195, GO:9143, GO:9144, GO:9261, GO:9199, GO:9166, GO:9141, GO:5794,		IGL@, GGTLC2, RTDR1, GNAZ, RAB36, BCR,
ASD	22	21995356	22598120	Dup	Inherited	No	No	GO:6633, GO:6461,		IGLL1, C22orf43, RGL4, ZNF70, VPREB3, C22orf15, CHCHD10, MMP11, SMARCB1, DERL3, SLC2A11, MIF,
ASD	22	22641474	23323367	Dup	Inherited	No	No	GO:6633, GO:51301,	SPECC1L,	DDTL, DDT, GSTT2, LOC391322, GSTT1, CABIN1, SUSD2, GGT5, SPECC1L, ADORA2A, UPB1, GUCD1, SNRPD3, GGT1, FAM211B,
ASD	22	47780315	48387485	Dup	Inherited	No	No			
ASD	22	47780315	48387485	Dup	Inherited	No	No			
ASD	23	3702747	4567265	Dup	Inherited	No	No			LOC729162,
ASD	23	6456825	8095053	Dup	Inherited	No	No	GO:5794,		VCX3A, HDHD1, STS, VCX, PNPLA4,
ASD	23	6456825	8095053	Dup	Inherited	No	No	GO:5794,		VCX3A, HDHD1, STS, VCX, PNPLA4,
ASD	23	9931816	10758861	Dup	Inherited	No	No			WWC3, CLCN4, MID1,
ASD	23	24087361	24612313	Dup	Inherited	No	No			ZFX, SUPT20HL2, SUPT20HL1, PDK3, PCYT1B,
ASD	23	28931559	29478966	Dup	Inherited	No	No			IL1RAPL1,
ASD	23	88052525	88616963	Del	Inherited	No	No			
ASD	23	139846520	141067065	Dup	Inherited	No	No			SPANXB2, SPANXB1, LDOC1, SPANXC, SPANXA1, SPANXA2, SPANXD, MAGEC3, MAGEC1,
ASD	23	148075334	148617551	Dup	Inherited	No	No			IDS, CXorf40A, MAGEA9B, HSFX2, TMEM185A, MAGEA11,
ASD	7	61100583	62082083	Dup	Denovo	No	No			
ASD	8	704383	1521910	Dup	Denovo	No	No			DLGAP2,
ASD	8	65354366	66254869	Del	Denovo	No	No	GO:42493,	CYP7B1 ,	BHLHE22, CYP7B1,
ASD	8	66140254	66753892	Del	Denovo	No	No			ARMC1, MTFR1,
ASD	9	98998	3682923	Del	Denovo	No	No	GO:9154, GO:9205, GO:6195, GO:9143, GO:9144, GO:9261, GO:9199, GO:9166, GO:9141, GO:42493, GO:10927,	VLDLR,	FOXD4, CBWD1, C9orf66, DOCK8, KANK1, DMRT1, DMRT3, DMRT2, SMARCA2, VLDLR, KCNV2, KIAA0020, RFX3,
ASD	9	138237996	139334064	Dup	Denovo	No	No	GO:5794, GO:42493, GO:51301, GO:5516, GO:6461, GO:6633, GO:9154, GO:9205, GO:6195, GO:9143, GO:9144, GO:9261, GO:9199, GO:9166, GO:9141, GO:48285, PAN-PW:16,	CARD9, NOTCH1 , ABCA2, ANAPC2 ,	QSOX2, GPSM1, DNLZ, CARD9, SNAPC4, SDCCAG3, PMPCA, INPP5E, SEC16A, C9orf163, NOTCH1, EGFL7, AGPAT2, FAM69B, LCN10, LCN6, LCN8, LCN15, TMEM141, KIAA1984, RABL6, C9orf172, PHPT1, MAMDC4, EDF1, TRAF2, FBXW5, C8G, LCN12, PTGDS, LCNL1, C9orf142, CLIC3, ABCA2, C9orf139, FUT7, NPDC1, ENTPD2, SAPCD2, UAP1L1, MAN1B1, DPP7, GRIN1, LRRC26, ANAPC2, SSNA1, TPRN, TMEM203, NDOR1, RNF208, RNF224, SLC34A3, TUBB4B, FAM166A, C9orf173, COBRA1, TOR4A, NRARP, EXD3,
ASD	9	138505259	139336068	Dup	Denovo	No	No	GO:5794, GO:51301, GO:5516, GO:6461, GO:6633, GO:42493, GO:9154, GO:9205, GO:6195, GO:9143, GO:9144, GO:9261, GO:9199, GO:9166, GO:9141, GO:48285, PAN-PW:16,	NOTCH1 , ABCA2, ANAPC2 ,	NOTCH1, EGFL7, AGPAT2, FAM69B, LCN10, LCN6, LCN8, LCN15, TMEM141, KIAA1984, RABL6, C9orf172, PHPT1, MAMDC4, EDF1, TRAF2, FBXW5, C8G, LCN12, PTGDS, LCNL1, C9orf142, CLIC3, ABCA2, C9orf139, FUT7, NPDC1, ENTPD2, SAPCD2, UAP1L1, MAN1B1, DPP7, GRIN1, LRRC26, ANAPC2, SSNA1, TPRN, TMEM203, NDOR1, RNF208, RNF224, SLC34A3, TUBB4B, FAM166A, C9orf173, COBRA1, TOR4A, NRARP, EXD3,
ASD	12	54218922	58779615	Dup	Denovo	No	No	GO:5794, GO:51301, GO:48285, GO:9154, GO:9205, GO:6195, GO:9143, GO:9144, GO:9261, GO:9199, GO:9166, GO:9141, GO:42493, GO:6633, GO:5516, PAN-PW:44, GO:6461,	CDK2, ERBB3, TIMELESS, MYO1A, CDK4,	OR6C4, OR2AP1, OR10P1, METTL7B, ITGA7, BLOC1S1, RDH5, CD63, GDF11, SARNP, ORMDL2, DNAJC14, MMP19, WIBG, DGKA, PMEL, CDK2, RAB5B, SUOX, IKZF4, RPS26, ERBB3, PA2G4, RPL41, ZC3H10, ESYT1, MYL6B, MYL6, SMARCC2, RNF41, NABP2, SLC39A5, ANKRD52, COQ10A, CS, CNPY2, PAN2, IL23A, STAT2, APOF, TIMELESS, MIP, SPRYD4, GLS2, RBMS2, BAZ2A, ATP5B, PTGES3, NACA, PRIM1, HSD17B6, SDR9C7, RDH16, GPR182, ZBTB39, TAC3, MYO1A, TMEM194A, NAB2, STAT6, LRP1, NXPH4, SHMT2, NDUFA4L2, STAC3, R3HDM2, INHBC, INHBE, GLI1, ARHGAP9, MARS, DDIT3, MBD6, DCTN2, KIF5A, PIP4K2C, DTX3, ARHGEF25, SLC26A10, B4GALNT1, OS9, AGAP2, TSPAN31, CDK4, MARCH-9, CYP27B1, METTL1, METTL21B, TSFM, AVIL, CTDSP2, XRCC6BP1, LRIG3, SLC16A7,
ASD	15	21190624	26203954	Dup	Denovo	Yes	Yes	GO:9154, GO:9205, GO:6195, GO:9143, GO:9144, GO:9261, GO:9199, GO:9166, GO:9141, GO:42493,	GABRG3 ,	GOLGA6L2, MKRN3, MAGEL2, NDN, NPAP1, SNRPN, SNURF, UBE3A, ATP10A, GABRB3, GABRA5, GABRG3, OCA2, HERC2,
ASD	16	29502984	30210849	Dup	Denovo	Yes	Yes	GO:6461, GO:5794, GO:48285, GO:42493, GO:9205, GO:9144, GO:9199, GO:9141,	MVP,	SPN, QPRT, C16orf54, ZG16, KIF22, MAZ, PRRT2, MVP, PAGR1, CDIPT, SEZ6L2, ASPHD1, KCTD13, TMEM219, TAOK2, HIRIP3, INO80E, DOC2A, C16orf92, FAM57B, ALDOA, PPP4C, TBX6, YPEL3, GDPD3, MAPK3, CORO1A, BOLA2B, SLX1A, SULT1A3,
ASD	16	29502984	30127026	Del	Denovo	Yes	Yes	GO:6461, GO:5794, GO:48285, GO:42493, GO:9205, GO:9144, GO:9199, GO:9141,	MVP,	SPN, QPRT, C16orf54, ZG16, KIF22, MAZ, PRRT2, MVP, PAGR1, CDIPT, SEZ6L2, ASPHD1, KCTD13, TMEM219, TAOK2, HIRIP3, INO80E, DOC2A, C16orf92, FAM57B, ALDOA, PPP4C, TBX6, YPEL3, GDPD3, MAPK3, CORO1A, BOLA2B, SLX1A, SULT1A3,
ASD	16	29554843	30195224	Del	Denovo	Yes	Yes	GO:6461, GO:5794, GO:48285, GO:42493, GO:9205, GO:9144, GO:9199, GO:9141,	MVP,	SPN, QPRT, C16orf54, ZG16, KIF22, MAZ, PRRT2, MVP, PAGR1, CDIPT, SEZ6L2, ASPHD1, KCTD13, TMEM219, TAOK2, HIRIP3, INO80E, DOC2A, C16orf92, FAM57B, ALDOA, PPP4C, TBX6, YPEL3, GDPD3, MAPK3, CORO1A, BOLA2B, SLX1A, SULT1A3,
ASD	17	76953064	77782267	Dup	Denovo	No	No	GO:10927, PAN-PW:16, PAN-PW:44, GO:51301, GO:48285, GO:9154, GO:9205, GO:6195, GO:9143, GO:9144, GO:9261, GO:9199, GO:9166, GO:9141, GO:6461, GO:5794, GO:6633,	ACTG1, ANAPC11,	LOC100130370, BAHCC1, ACTG1, FSCN2, C17orf70, NPLOC4, TSPAN10, PDE6G, OXLD1, CCDC137, ARL16, HGS, MRPL12, SLC25A10, PPP1R27, P4HB, ARHGDIA, ALYREF, ANAPC11, NPB, PCYT2, SIRT7, MAFG, PYCR1, MYADML2, NOTUM, ASPSCR1, STRA13, LRRC45, RAC3, DCXR, RFNG, GPS1, DUS1L, FASN, CCDC57, SLC16A3,
ADHD	1	5198919	5747986	Dup	Unknown	No	No			
ADHD	1	5220394	5754188	Dup	Unknown	No	No			
ADHD	1	53772097	54338898	Dup	Unknown	No	No	GO:6461, GO:5794,		GLIS1, TMEM48, YIPF1, DIO1, HSPB11, LRRC42, LDLRAD1, TMEM59, TCEANC2,
ADHD	1	144975398	146268315	Dup	Unknown	No	Yes	GO:9154, GO:9205, GO:6195, GO:9143, GO:9144, GO:9261, GO:9199, GO:9166, GO:9141, GO:5794,		PRKAB2, FMO5, CHD1L, BCL9, ACP6, GJA5, GJA8, GPR89B, NBPF24,
ADHD	1	159732044	160485312	Del	Unknown	No	No	GO:5794,		FCGR2A, HSPA6, FCGR3A, FCGR2C, FCGR3B, FCGR2B, FCRLA, FCRLB, DUSP12, ATF6, OLFML2B, NOS1AP,
ADHD	1	235967993	237757467	Dup	Unknown	No	No	GO:5516, MGI:5620,		RYR2, ZP4,
ADHD	1	245714416	246522135	Del	Unknown	No	No			OR2W5, OR2C3, GCSAML, OR2G2, OR2G3, OR13G1, OR6F1, OR1C1, OR14A16, OR11L1, TRIM58, OR2W3, OR2T8, OR2L13, OR2L8, OR2AK2, OR2L5, OR2L2, OR2L3, OR2M5, OR2M2, OR2M3, OR2M4, OR2T33,
ADHD	2	32487194	33181898	Dup	Unknown	No	Yes			BIRC6, TTC27, LTBP1,
ADHD	2	32497032	33181898	Dup	Unknown	No	Yes			BIRC6, TTC27, LTBP1,
ADHD	2	106690781	108292465	Dup	Unknown	No	No	GO:5794,		ST6GAL2, RGPD4, SLC5A7, SULT1C3, SULT1C2,
ADHD	2	107907419	109818614	Del	Unknown	No	No	GO:5794, GO:48285, GO:51301,	SEPT-10,	SLC5A7, SULT1C3, SULT1C2, SULT1C4, GCC2, LIMS1, RANBP2, CCDC138, EDAR, SH3RF3, SEPT-10, SOWAHC,
ADHD	2	128985135	129881989	Del	Unknown	No	No			
ADHD	2	153846483	154652950	Dup	Unknown	No	No	GO:5794,		RPRM, GALNT13,
ADHD	3	849785	1451706	Dup	Unknown	No	Yes			CNTN6,
ADHD	3	1166638	2183832	Dup	Unknown	Yes	Yes			CNTN6, CNTN4,
ADHD	3	2969491	4461303	Del	Unknown	Yes	No	GO:9154, GO:9205, GO:6195, GO:9143, GO:9144, GO:9261, GO:9199, GO:9166, GO:9141,		CNTN4, IL5RA, TRNT1, CRBN, LRRN1, SETMAR, SUMF1,
ADHD	3	3999657	4922174	Dup	Unknown	Yes	No			SETMAR, SUMF1, ITPR1,
ADHD	3	32039611	33903472	Dup	Unknown	No	No	GO:51301, GO:48285, GO:5794,	DYNC1LI1, CLASP2 , PDCD6IP,	GPD1L, CMTM8, CMTM7, CMTM6, DYNC1LI1, CNOT10, TRIM71, CCR4, GLB1, TMPPE, CRTAP, SUSD5, FBXL2, UBP1, CLASP2, PDCD6IP,
ADHD	3	96242194	98639625	Dup	Unknown	No	Yes			EPHA6,
ADHD	3	174547344	176016137	Dup	Unknown	Yes	No	GO:6461,		NLGN1,
ADHD	3	193473269	194025326	Dup	Unknown	No	No			FGF12, MB21D2,
ADHD	4	18233904	19901337	Dup	Unknown	No	No			SLIT2,
ADHD	4	23596065	27014659	Dup	Unknown	No	No	GO:9154, GO:9205, GO:6195, GO:9143, GO:9144, GO:9261, GO:9199, GO:9166, GO:9141, GO:5794, GO:51301, GO:48285,	ANAPC4 ,	LOC729175, DHX15, SOD3, CCDC149, LGI2, SEPSECS, PI4K2B, ZCCHC4, ANAPC4, SLC34A2, SEL1L3, C4orf52, RBPJ, CCKAR, TBC1D19, STIM2,
ADHD	4	26350218	27635050	Dup	Unknown	No	No			TBC1D19, STIM2,
ADHD	4	65791505	66605654	Del	Unknown	No	No			EPHA5,
ADHD	4	71457385	72257388	Dup	Unknown	No	No			AMBN, ENAM, IGJ, UTP3, RUFY3, GRSF1, MOB1B, DCK,
ADHD	4	75931128	77058132	Dup	Unknown	No	Yes	GO:5794,		BTC, PARM1, RCHY1, THAP6, C4orf26, CDKL2, G3BP2, USO1, PPEF2, NAAA,
ADHD	4	89231344	90528335	Dup	Unknown	No	No	GO:42493, GO:9154, GO:9205, GO:6195, GO:9143, GO:9144, GO:9261, GO:9199, GO:9166, GO:9141,	ABCG2,	ABCG2, PPM1K, HERC6, HERC5, PIGY, HERC3, NAP1L5, FAM13A, LOC731282, TIGD2, GPRIN3,
ADHD	4	184952586	185493309	Dup	Unknown	No	No			STOX2, ENPP6, LOC391722,
ADHD	4	189649974	190598641	Del	Unknown	No	Yes			
ADHD	6	122085305	122622855	Dup	Unknown	No	No			
ADHD	7	17811400	18312755	Dup	Unknown	No	No			SNX13, PRPS1L1,
ADHD	7	69777660	71572992	Dup	Unknown	Yes	No	GO:5794,		AUTS2, WBSCR17, CALN1,
ADHD	7	122788319	123645842	Dup	Unknown	No	No	GO:6461, GO:5794, PAN-PW:16,		IQUB, NDUFA5, ASB15, LMOD2, WASL, HYAL4, SPAM1, TMEM229A,
ADHD	8	5374826	6304573	Del	Unknown	No	No			MCPH1,
ADHD	8	9082416	9659969	Dup	Unknown	No	No	GO:5794,		TNKS,
ADHD	8	18578565	19386565	Dup	Unknown	No	Yes	GO:5794,		PSD3, SH2D4A, CSGALNACT1,
ADHD	8	43143782	43910848	Dup	Unknown	No	No	GO:6461,		HGSNAT, POTEA,
ADHD	8	85438801	88424995	Del	Unknown	No	No	GO:51301, GO:48285, GO:5794, MGI:5620,	LRRCC1 ,	RALYL, LRRCC1, E2F5, C8orf59, CA13, CA1, CA3, CA2, REXO1L1, PSKH2, ATP6V0D2, SLC7A13, WWP1, FAM82B, CPNE3, CNGB3, CNBD1,
ADHD	8	89227035	90419753	Dup	Unknown	No	Yes			MMP16,
ADHD	10	47920669	50764666	Del	Unknown	No	No	MGI:5620, GO:9154, GO:9205, GO:6195, GO:9143, GO:9144, GO:9261, GO:9199, GO:9166, GO:9141, GO:42493, PAN-PW:44, GO:5794,	CHAT, SLC18A3,	ZNF488, RBP3, GDF2, GDF10, PTPN20B, FAM25C, FRMPD2, MAPK8, ARHGAP22, WDFY4, LRRC18, VSTM4, FAM170B, C10orf128, C10orf71, DRGX, ERCC6, PGBD3, CHAT, SLC18A3, C10orf53, OGDHL,
ADHD	11	5582423	7488751	Del	Unknown	No	No	GO:6461, GO:5794, GO:42493, GO:10927,	SMPD1,	TRIM6, TRIM6-TRIM34, TRIM34, TRIM5, TRIM22, OR56B1, OR52N4, OR52N5, OR52N1, OR52N2, OR52E6, OR52E8, OR52E4, OR56A3, OR56A5, OR52L1, OR56A4, OR56A1, OR56B4, OR52B2, OR52W1, C11orf42, FAM160A2, CNGA4, CCKBR, PRKCDBP, SMPD1, APBB1, HPX, TRIM3, ARFIP2, TIMM10B, DNHD1, RRP8, ILK, TAF10, TPP1, DCHS1, MRPL17, OR2AG2, OR2AG1, OR6A2, OR10A5, OR10A2, OR10A4, OR2D2, OR2D3, ZNF215, ZNF214, NLRP14, RBMXL2, SYT9, OLFML1,
ADHD	11	59437619	60222063	Del	Unknown	No	No			PLAC1L, MS4A3, MS4A2, MS4A6A, MS4A4E, MS4A4A, MS4A6E, MS4A7, MS4A14, MS4A5, MS4A1, MS4A12, MS4A13,
ADHD	11	67841909	69060612	Dup	Unknown	No	No	GO:5794, GO:42493, GO:6461, GO:9154, GO:9205, GO:6195, GO:9143, GO:9144, GO:9261, GO:9199, GO:9166, GO:9141,	GAL, CPT1A,	LRP5, PPP6R3, GAL, MTL5, CPT1A, MRPL21, IGHMBP2, MRGPRD, TPCN2, MYEOV,
ADHD	11	102605727	106728114	Del	Unknown	No	No	GO:5794, GO:9154, GO:9205, GO:6195, GO:9143, GO:9144, GO:9261, GO:9199, GO:9166, GO:9141,		DYNC2H1, PDGFD, DDI1, CASP4, CASP5, CASP1, CARD16, CARD17, CARD18, GRIA4, MSANTD4, KBTBD3, AASDHPPT, GUCY1A2, CWF19L2,
ADHD	11	133553353	134382876	Dup	Unknown	No	No	GO:51301, GO:48285, GO:5794,	NCAPD3 ,	NCAPD3, VPS26B, THYN1, ACAD8, GLB1L3, GLB1L2, B3GAT1,
ADHD	12	28812958	29402567	Dup	Unknown	No	No	GO:5794,		FAR2, ERGIC2,
ADHD	12	33175713	33809963	Del	Unknown	No	No			SYT10,
ADHD	12	34122001	34711193	Dup	Unknown	No	No			
ADHD	13	22494127	23860983	Dup	Unknown	No	No			SGCG, SACS, TNFRSF19, MIPEP, C1QTNF9B-AS1, C1QTNF9B, SPATA13, C1QTNF9,
ADHD	13	63330923	63886321	Del	Unknown	No	No			
ADHD	14	92009962	92767834	Dup	Unknown	No	No	GO:5794,		SLC24A4, RIN3, LGMN, GOLGA5, CHGA, ITPK1, MOAP1, TMEM251, C14orf142, UBR7,
ADHD	15	21016722	25902239	Dup	Unknown	Yes	Yes	GO:9154, GO:9205, GO:6195, GO:9143, GO:9144, GO:9261, GO:9199, GO:9166, GO:9141, GO:42493,	GABRG3 ,	LOC440243, GOLGA6L2, MKRN3, MAGEL2, NDN, NPAP1, SNRPN, SNURF, UBE3A, ATP10A, GABRB3, GABRA5, GABRG3, OCA2,
ADHD	15	27000239	28153539	Dup	Unknown	No	Yes	GO:5794,		APBA2, NDNL2, TJP1,
ADHD	15	29722573	30302218	Dup	Unknown	No	Yes	PAN-PW:44,	CHRNA7 ,	OTUD7A, LOC100130857, CHRNA7,
ADHD	15	29722573	30302218	Dup	Unknown	No	Yes	PAN-PW:44,	CHRNA7 ,	OTUD7A, LOC100130857, CHRNA7,
ADHD	15	29722573	30302218	Dup	Unknown	No	Yes	PAN-PW:44,	CHRNA7 ,	OTUD7A, LOC100130857, CHRNA7,
ADHD	15	29722573	30302218	Dup	Unknown	No	Yes	PAN-PW:44,	CHRNA7 ,	OTUD7A, LOC100130857, CHRNA7,
ADHD	15	29734334	30302218	Dup	Unknown	No	Yes	PAN-PW:44,	CHRNA7 ,	OTUD7A, LOC100130857, CHRNA7,
ADHD	15	29734334	30302218	Dup	Unknown	No	Yes	PAN-PW:44,	CHRNA7 ,	OTUD7A, LOC100130857, CHRNA7,
ADHD	15	30688712	32587887	Dup	Unknown	No	No	MGI:5620, GO:5794,		ARHGAP11A, SCG5, GREM1, FMN1, RYR3, AVEN, CHRM5, EMC7, PGBD4, KATNBL1, EMC4, SLC12A6, NOP10, C15orf55, LPCAT4, GOLGA8A,
ADHD	15	30713368	32447708	Del	Unknown	No	No	MGI:5620,		ARHGAP11A, SCG5, GREM1, FMN1, RYR3, AVEN, CHRM5, EMC7, PGBD4, KATNBL1, EMC4, SLC12A6, NOP10, C15orf55, LPCAT4,
ADHD	15	31406234	31913133	Dup	Unknown	No	No	MGI:5620,		RYR3,
ADHD	15	82183518	82749322	Dup	Unknown	No	No			ADAMTSL3, GOLGA6L4,
ADHD	16	15032942	16197033	Dup	Unknown	No	Yes	GO:51301, GO:48285, GO:5516, GO:10927, GO:6461, PAN-PW:16, PAN-PW:44, MGI:5620, GO:5794, GO:42493, GO:9154, GO:9205, GO:6195, GO:9143, GO:9144, GO:9261, GO:9199, GO:9166, GO:9141, GO:6633,	NDE1, MYH11, ABCC1, ABCC6,	PDXDC1, NTAN1, RRN3, MPV17L, C16orf45, KIAA0430, NDE1, MYH11, FOPNL, ABCC1, ABCC6,
ADHD	16	15032942	16197033	Dup	Unknown	No	Yes	GO:51301, GO:48285, GO:5516, GO:10927, GO:6461, PAN-PW:16, PAN-PW:44, MGI:5620, GO:5794, GO:42493, GO:9154, GO:9205, GO:6195, GO:9143, GO:9144, GO:9261, GO:9199, GO:9166, GO:9141, GO:6633,	NDE1, MYH11, ABCC1, ABCC6,	PDXDC1, NTAN1, RRN3, MPV17L, C16orf45, KIAA0430, NDE1, MYH11, FOPNL, ABCC1, ABCC6,
ADHD	16	15156431	18174650	Dup	Unknown	No	Yes	GO:51301, GO:48285, GO:5516, GO:10927, GO:6461, PAN-PW:16, PAN-PW:44, MGI:5620, GO:5794, GO:42493, GO:9154, GO:9205, GO:6195, GO:9143, GO:9144, GO:9261, GO:9199, GO:9166, GO:9141, GO:6633,	NDE1, MYH11, ABCC1, ABCC6,	MPV17L, C16orf45, KIAA0430, NDE1, MYH11, FOPNL, ABCC1, ABCC6, NOMO3, XYLT1,
ADHD	16	15387380	16190572	Dup	Unknown	No	Yes	GO:51301, GO:48285, GO:5516, GO:10927, GO:6461, PAN-PW:16, PAN-PW:44, MGI:5620, GO:5794, GO:42493, GO:9154, GO:9205, GO:6195, GO:9143, GO:9144, GO:9261, GO:9199, GO:9166, GO:9141, GO:6633,	NDE1, MYH11, ABCC1, ABCC6,	MPV17L, C16orf45, KIAA0430, NDE1, MYH11, FOPNL, ABCC1, ABCC6,
ADHD	16	15387380	16197033	Dup	Unknown	No	Yes	GO:51301, GO:48285, GO:5516, GO:10927, GO:6461, PAN-PW:16, PAN-PW:44, MGI:5620, GO:5794, GO:42493, GO:9154, GO:9205, GO:6195, GO:9143, GO:9144, GO:9261, GO:9199, GO:9166, GO:9141, GO:6633,	NDE1, MYH11, ABCC1, ABCC6,	MPV17L, C16orf45, KIAA0430, NDE1, MYH11, FOPNL, ABCC1, ABCC6,
ADHD	16	15387380	16197033	Dup	Unknown	No	Yes	GO:51301, GO:48285, GO:5516, GO:10927, GO:6461, PAN-PW:16, PAN-PW:44, MGI:5620, GO:5794, GO:42493, GO:9154, GO:9205, GO:6195, GO:9143, GO:9144, GO:9261, GO:9199, GO:9166, GO:9141, GO:6633,	NDE1, MYH11, ABCC1, ABCC6,	MPV17L, C16orf45, KIAA0430, NDE1, MYH11, FOPNL, ABCC1, ABCC6,
ADHD	16	15398985	18174650	Dup	Unknown	No	Yes	GO:51301, GO:48285, GO:5516, GO:10927, GO:6461, PAN-PW:16, PAN-PW:44, MGI:5620, GO:5794, GO:42493, GO:9154, GO:9205, GO:6195, GO:9143, GO:9144, GO:9261, GO:9199, GO:9166, GO:9141, GO:6633,	NDE1, MYH11, ABCC1, ABCC6,	MPV17L, C16orf45, KIAA0430, NDE1, MYH11, FOPNL, ABCC1, ABCC6, NOMO3, XYLT1,
ADHD	16	15408600	16190572	Dup	Unknown	No	Yes	GO:51301, GO:48285, GO:5516, GO:10927, GO:6461, PAN-PW:16, PAN-PW:44, MGI:5620, GO:5794, GO:42493, GO:9154, GO:9205, GO:6195, GO:9143, GO:9144, GO:9261, GO:9199, GO:9166, GO:9141, GO:6633,	NDE1, MYH11, ABCC1, ABCC6,	MPV17L, C16orf45, KIAA0430, NDE1, MYH11, FOPNL, ABCC1, ABCC6,
ADHD	16	21851681	22651534	Del	Unknown	No	Yes	GO:5516,		UQCRC2, PDZD9, C16orf52, VWA3A, EEF2K, POLR3E, CDR2, LOC100132247,
ADHD	16	26396505	27321840	Dup	Unknown	No	No			C16orf82, KDM8, NSMCE1, IL4R, IL21R,
ADHD	16	29563594	30367556	Del	Unknown	Yes	Yes	GO:6461, GO:5794, GO:48285, GO:42493, GO:9205, GO:9144, GO:9199, GO:9141, GO:51301, GO:9143, GO:9166,	MVP, SEPT-1 ,	SPN, QPRT, C16orf54, ZG16, KIF22, MAZ, PRRT2, MVP, PAGR1, CDIPT, SEZ6L2, ASPHD1, KCTD13, TMEM219, TAOK2, HIRIP3, INO80E, DOC2A, C16orf92, FAM57B, ALDOA, PPP4C, TBX6, YPEL3, GDPD3, MAPK3, CORO1A, BOLA2B, SLX1A, SULT1A3, CD2BP2, TBC1D10B, MYLPF, SEPT-1, ZNF48, ZNF771, DCTPP1, SEPHS2,
ADHD	16	45096893	48052382	Dup	Unknown	No	No	GO:10927, PAN-PW:16, MGI:5620, GO:5516, GO:9154, GO:9205, GO:6195, GO:9143, GO:9144, GO:9261, GO:9199, GO:9166, GO:9141,		SHCBP1, VPS35, ORC6, MYLK3, C16orf87, GPT2, DNAJA2, NETO2, ITFG1, PHKB, ABCC12, ABCC11, LONP2, SIAH1, N4BP1, CBLN1, LOC100130314, C16orf78,
ADHD	16	85987740	86536663	Del	Unknown	No	No			MAP1LC3B, ZCCHC14, JPH3, KLHDC4, SLC7A5, CA5A,
ADHD	17	51088	583572	Dup	Unknown	No	No	GO:42493, GO:5794,	RPH3AL ,	RPH3AL, C17orf97, FAM101B, VPS53, LOC100129974, FAM57A,
ADHD	17	31889664	33319881	Dup	Unknown	No	No	PAN-PW:44, GO:5794, GO:51301, GO:6633, GO:42493, GO:6461, GO:9154, GO:9205, GO:6195, GO:9143, GO:9144, GO:9261, GO:9199, GO:9166, GO:9141,	MYO19, AATF, ACACA,	ZNHIT3, MYO19, PIGW, GGNBP2, DHRS11, MRM1, LHX1, AATF, ACACA, C17orf78, TADA2A, DUSP14, SYNRG, LOC100131822, DDX52, HNF1B,
ADHD	17	31964475	33297438	Dup	Unknown	No	No	PAN-PW:44, GO:5794, GO:51301, GO:6633, GO:42493, GO:6461, GO:9154, GO:9205, GO:6195, GO:9143, GO:9144, GO:9261, GO:9199, GO:9166, GO:9141,	MYO19, AATF, ACACA,	MYO19, PIGW, GGNBP2, DHRS11, MRM1, LHX1, AATF, ACACA, C17orf78, TADA2A, DUSP14, SYNRG, LOC100131822, DDX52, HNF1B,
ADHD	17	31979521	33318471	Del	Unknown	No	No	GO:5794, GO:51301, GO:6633, GO:42493, GO:6461, GO:9154, GO:9205, GO:6195, GO:9143, GO:9144, GO:9261, GO:9199, GO:9166, GO:9141,	AATF, ACACA,	GGNBP2, DHRS11, MRM1, LHX1, AATF, ACACA, C17orf78, TADA2A, DUSP14, SYNRG, LOC100131822, DDX52, HNF1B,
ADHD	19	23164768	23816927	Dup	Unknown	No	No			ZNF91, ZNF675, ZNF681,
ADHD	19	32615675	33220146	Del	Unknown	No	Yes			
ADHD	20	12795614	14242196	Dup	Unknown	No	No	GO:6461,		SPTLC3, ISM1, TASP1, ESF1, NDUFAF5, SEL1L2, MACROD2,
ADHD	22	17257787	19792353	Dup	Unknown	No	Yes	GO:5794, GO:48285, GO:51301, GO:9154, GO:9205, GO:6195, GO:9143, GO:9144, GO:9261, GO:9199, GO:9166, GO:9141, GO:42493, PAN-PW:44, GO:6461,	SEPT-5 , COMT, KLHL22 , SNAP29 ,	DGCR6, PRODH, LOC402036, DGCR2, DGCR14, TSSK2, GSC2, SLC25A1, CLTCL1, HIRA, MRPL40, C22orf39, UFD1L, CDC45, CLDN5, SEPT-5, GP1BB, TBX1, GNB1L, C22orf29, TXNRD2, COMT, ARVCF, C22orf25, DGCR8, TRMT2A, RANBP1, ZDHHC8, LOC388849, RTN4R, DGCR6L, TMEM191B, RIMBP3, LOC729461, USP41, ZNF74, SCARF2, KLHL22, MED15, PI4KA, SERPIND1, SNAP29, CRKL, AIFM3, LZTR1, THAP7, P2RX6, SLC7A4,

Note: This table includes the CNV type (deletion or duplication), inheritance status (inherited or de novo), where known, and whether the CNV overlaps known autism regions or case CNVs from the other disorder. The contribution of the CNVs to the top 20 pathways from Table 2 is shown, as are the genes that they hit. del = deletion; dup = duplication.
